# Tubular NAT10 Promotes the Secretion of TGF-β and Lactate to Drive Fibroblast Activation in Diabetic Kidney Disease

**DOI:** 10.7150/ijbs.140281

**Published:** 2026-09-02

**Authors:** Jinfang Lu, Yuqing Feng, Xujun Peng, Zijin He, Zhou Li, Ao Jia, Yaohui Bao, Lingui Xie, Renchun Wang, Wenbo Zhang, Qin Kang, Yanyan Zhou, Cheng Wang, Hongquan Peng, Huiling Li, Dongshan Zhang

**Affiliations:** 1 Department of Critical Care Medicine, The Second Xiangya Hospital, Central South University, Changsha, Hunan 410011, China.; 2 Department of Ophthalmology, The Second Xiangya Hospital, Central South University, Changsha, Hunan 410011, China.; 3 Hunan Clinical Research Center of Ophthalmic Disease, Changsha, Hunan 410011, China.; 4 Universiti Sains Malaysia, Kubang Kerian, Kelantan 16150, Malaysia.; 5 Furong Laboratory, Xiangya School of Medicine, Central South University, Changsha, Hunan 410004, China.; 6 Department of Biology and Medicine, Central South University of Forestry and Technology, Changsha, Hunan 410004, China.; 7 Hunan Provincial Center for Critical Care Medicine and Clinical Research in Smart Healthcare, Changsha, Hunan 410011, China.; 8 Division of Nephrology, Department of Medicine, the Fifth Affiliated Hospital of Sun Yat-Sen University, Zhuhai, Guangdong 519000, China.; 9 Guangdong-Hong Kong-Macao University Joint Laboratory of Interventional Medicine, The Fifth Affiliated Hospital of Sun Yat-Sen University, Zhuhai, Guangdong 519000, China.; 10 Department of Nephrology, Kiang Wu Hospital, Macau SAR 999078, China.

**Keywords:** diabetic kidney disease, tubulointerstitial fibrosis, NAT10, ac4C modification, lactate, TGF-β signaling

## Abstract

In advanced diabetic kidney disease (DKD), tubulointerstitial fibrosis (TIF) is a key histopathological lesion accompanying progressive renal functional decline. However, the tubular mechanisms that drive tubulointerstitial fibrogenesis remain incompletely understood. N-acetyltransferase 10 (NAT10) mediates mRNA N4-acetylcytidine (ac4C) modification, but its role and therapeutic potential in DKD are unknown. We investigated NAT10 regulation and function using cultured tubular cells, diabetic mouse models, patient kidney specimens, and tubule-specific Nat10 knockout mice, together with pharmacological treatment using guanosine diphosphate disodium salt. High glucose or diabetes increased tubular NAT10 abundance in cultured cells, mouse kidneys, and human DKD tissues through activation of NF-κB signaling. Mechanistically, NAT10 in tubular cells stabilized *Brd4* and *Pfkm* mRNAs through ac4C modification, activating the STAT3/TGF-β and lactate/H3K18 lactylation pathways. These pathways synergistically promoted fibroblast activation through NOTCH/SMAD3 signaling and formed a positive feedback loop with NF-κB. Tubule-specific *Nat10* deletion reduced *Brd4* and *Pfkm* expression, suppressed the associated signaling cascades, and mitigated kidney fibrosis in the DKD model. Consistently, guanosine diphosphate disodium salt alleviated renal injury and fibrosis in *db/db* mice. Thus, NAT10 drives tubular fibrosis through ac4C-dependent profibrotic signaling, supporting NAT10 inhibition as a potential therapeutic strategy for DKD.

## Introduction

Diabetes has emerged as a substantial worldwide public health burden across countries at different stages of economic development. The number of individuals living with diabetes is predicted to increase to 643 million by 2030^1^. Diabetic kidney disease (DKD) is a common long-term complication affecting individuals with either type 1 or type 2 diabetes and is widely recognized as a major contributor to chronic kidney disease (CKD) and end-stage kidney disease requiring renal replacement therapy worldwide^1^-^3^. It is estimated that roughly 30% to 40% of diabetic patients develop DKD throughout their lifetime 4, 5.

DKD pathogenesis encompasses glomerular hemodynamic disturbances, tubulointerstitial dysfunction, and fibrosis 6, 7. However, the mechanisms that drive tubulointerstitial damage during DKD progression remain largely unclear. Tubulointerstitial fibrosis represents a primary pathological characteristic of CKD and encompasses excessive extracellular matrix accumulation within the renal interstitium. In addition, communication between tubular and interstitial compartments is increasingly recognized as an important mechanism promoting renal fibrotic progression^8^, ^9^. Earlier research has shown that PRDM16 within tubular cells enhances cytokine production, including TGF-β, thereby activating fibroblasts 10-12. More recent findings have shown that *Mettl3* in tubular cells promotes lactate secretion, thereby driving fibroblast activation 13. However, the common upstream mechanisms responsible for the secretion of TGF-β and lactate, as well as the potential presence and nature of any synergistic interaction between them, remain unclear.

RNA modifications constitute essential regulatory mechanisms governing RNA metabolism and gene expression, thereby maintaining cellular homeostasis, and they serve pivotal regulatory functions in a variety of diseases 14, 15. N6-methyladenosine (m6A), 5-methylcytidine (m5C), N1-methyladenosine (m1A), N7-methylguanosine (m7G), and N4-acetylcytidine (ac4C) are common RNA modifications in cells 16, 17. N4-acetylcytidine (ac4C) modification has been widely identified in multiple RNA species, including tRNAs, rRNAs, and mRNAs 18, 19, and is evolutionarily conserved in both prokaryotes and eukaryotes 20. It has been reported that ac4C is mainly distributed within the coding sequence (CDS) regions of human HeLa cells^18^, and its principal function in mRNA modification is to enhance mRNA stability and translational efficiency 18, 21, 22. Aberrant ac4C levels have been observed in multiple metabolic disorders and cancers 22-24. Nevertheless, the specific function of ac4C modification in renal fibrosis has not yet been elucidated. N-acetyltransferase 10 (NAT10), a conserved enzyme of the general control nonrepressible 5-related N-acetyltransferase (GNAT) family, possesses histone acetyltransferase activity and catalyzes ac4C formation across species^23^. This enzyme has been linked to multiple cellular events and disease-related processes, including cell-cycle regulation, autophagy, rRNA processing, microtubule stability, and senescence^25^-^28^. Emerging evidence indicates that RNA ac4C modification mediated by NAT10 participates in fibrosis of cardiac, hepatic, and pulmonary tissues 29-32. Nevertheless, whether NAT10 contributes to renal fibrogenesis in DKD remains unclear. Therefore, this study was designed to define the link between NAT10 expression and DKD-associated clinical and pathological features, and to clarify how NAT10-dependent ac4C regulation contributes to renal fibrosis during disease progression. Furthermore, it was demonstrated that NAT10 in tubular cells may promote ac4C modification of *Brd4* and *Pfkm* mRNA, thereby sequentially activating the STAT3/TGF-β pathway and the PFKM/lactate pathway. Ultimately, TGF-β and lactate jointly promote fibroblast activation through the NOTCH/SMAD3 axis. Meanwhile, the successful attenuation of DKD-associated renal fibrosis by guanosine diphosphate (GDP) disodium salt *in vitro* and *in vivo* indicates that NAT10 may function as a promising therapeutic target for DKD, and that GDP disodium salt may represent a potential therapeutic compound.

## Materials and Methods

### Antibodies and reagents

Primary antibodies against NAT10 (ab194297), ac4C (ab252215), fibronectin (ab2413), collagen IV (ab6586; for immunohistochemistry), and histone H3 (ab1791) were purchased from Abcam (Cambridge, UK). Antibodies targeting PFKM (55028-1-AP), NOTCH1 (10062-2-AP), α-SMA (14395-1-AP), collagen I (67288-1-Ig), and TGF-β1 (81746-2-RR) were supplied by Proteintech (Rosemont, IL, USA). Antibodies recognizing STAT3 (12640S), phospho-STAT3 (9145S), phospho-p65 (3033S), p65 (8242T), SMAD3 (9523S), and phospho-SMAD3 (9520S) were purchased from Cell Signaling Technology (Danvers, MA, USA). Anti-BRD4 (DF2905) and anti-β-tubulin (T2003) were from Affinity (Las Vegas, USA), and anti-Collagen IV used for immunoblotting (YT5768) was from Immunoway (San Jose, CA, USA). Anti-H3K18lac (PTM-1406RM) was supplied by PTM Bio (Hangzhou, China). Secondary antibodies were from Affinity. *Brd4*-targeting siRNA and control siRNA were supplied by RiboBio (Guangzhou, China), while *Brd4* and *Pfkm* expression plasmids were generated by Genechem (Shanghai, China). GDP disodium salt (HY-113066A) and BAY 11-7082 were purchased from MedChemExpress China (Shanghai, China). Streptozotocin was from Sigma Chemical Co. (St. Louis, MO, USA). The NAT10 ELISA kit and lactic acid assay kit (A019-2-1) were obtained from MLBIO (Shanghai, China) and Jiancheng Biotechnique (Nanjing, China), respectively.

### Cell culture and treatments

BUMPT cells (Boston University Mouse Proximal Tubule cells), derived from mouse proximal tubules, were provided by Dr. Wilfred Lieberthal from Boston University School of Medicine. These cells were maintained at 37 °C in a humidified 5% CO₂ atmosphere using DMEM (Gibco, 11965092) containing 10% fetal bovine serum (Gibco, 10100147) and 1% penicillin-streptomycin (Gibco, 15140163)^33^. To modulate specific gene expression, siRNA (5 ng) or plasmid (2 μg) was introduced into cells via Lipofectamine 2000 (PA530492, Invitrogen, MA, USA) per the supplier's protocols. Twenty-four hours later, cultures were exposed for 24-48 h to normal glucose (NG, 5 mM D-glucose), high glucose (HG, 30 mM D-glucose), or an osmotic control containing 5 mM glucose plus 25 mM D-mannitol.

Stable BUMPT lines with reduced or increased *Nat10* expression were generated as described previously 33. Briefly, Genechem (Shanghai, China) synthesized the *Nat10* expression vector containing the promoter, selection cassette, and cloning elements. The construct was introduced into cells by liposome-based transfection, after which puromycin-resistant clones were selected. Successful *Nat10* knockdown or overexpression was confirmed by PCR, immunoblotting, and fluorescence microscopy.

### Animal models

Male C57BL/6J mice, *db/db* mice, and their non-diabetic controls(*db/m*) were obtained from Hunan SJA Laboratory Animal Company. Mice were maintained in an SPF barrier facility with controlled temperature conditions of 22-25 °C and a regulated light-dark schedule, and were provided standard chow and water ad libitum. All experimental procedures were approved by the Animal Care and Use Ethics Committee of Second Xiangya Hospital, China (Approval No. 20260728).

To examine the in vivo role of NAT10 protein, conditional knockout mice with renal tubule-specific deletion of *Nat10* (T-NAT10-KO mice) were generated (Figure S2A, Supplementary Material). Genotypes were confirmed by three independent PCR assays for each mouse. (Figure S2B, Supplementary Material).

### Mouse models of diabetes

To establish a diabetic mouse model, 8-10-week-old male mice were weight-matched and randomly divided into a control (Ctrl) group and a streptozotocin (STZ)-treated diabetic group. Diabetes was induced by daily intraperitoneal injection of STZ (50 mg/kg body weight) dissolved in 50 mmol/L sodium citrate (SC) buffer for 5 consecutive days. Concurrently, an equal volume of SC buffer was administered to mice in the control group via the same injection route^34^. Diabetes was confirmed when blood glucose levels exceeded 200 mg/dL in two consecutive measurements. To monitor disease progression, body weight and blood glucose levels were measured weekly in diabetic mice from 8 to 18 weeks after model establishment, and the SC group was followed in parallel for 18 weeks. Urinary albumin and creatinine concentrations were quantified using established protocols, followed by calculation of the albumin-to-creatinine ratio (ACR)^35^. At the study endpoint, kidneys were collected after removing the renal capsules^36^.

A subset of kidney tissues underwent fixation and processing for pathological evaluation, and then subjected to histological staining including hematoxylin and eosin (H&E) staining, Masson's trichrome (MT) staining, as well as immunohistochemistry (IHC). An additional portion was retained for protein analysis via Western blot. Eight-week-old diabetic *db/db* mice and non-diabetic *db/m* mice received intraperitoneal administration of GDP disodium salt (10 mg/kg) or an equal volume of phosphate-buffered saline (PBS) as the vehicle control once every 3 days for 10 consecutive weeks. After ten weeks, the animals underwent euthanization, and their kidneys were collected for subsequent analyses. Six mice were included in each experimental group. The number of animals per group was selected based on previously reported effect sizes from DKD mouse model studies^37^.

### Patients and tissue samples

Ethical approval for this study was granted by the Ethics Review Board of the Fifth Affiliated Hospital of Sun Yat-sen University, Zhuhai, Guangdong, China (Approval No. 2025K24-1). All participants provided written informed consent prior to enrollment. From February 2025 to February 2026, urine, serum, and renal biopsy specimens were obtained from individuals diagnosed with DKD or renal cell carcinoma at this hospital. The cohort comprised 25 patients with DKD (13 early-stage and 12 late-stage) and 9 patients with renal cell carcinoma; detailed clinical characteristics are provided in Supplementary Table S1. Kidney biopsy specimens underwent fixation in 4% buffered paraformaldehyde before processing for H&E staining, MT staining, and IHC analysis following established protocols^10^, ^24^, ^38^. The pathological control (PC) group consisted of patients with histologically confirmed adjacent non-tumor renal tissues collected in the context of renal carcinoma.

The enrollment criteria for the DKD group were defined as follows: (1) type 2 diabetes mellitus; (2) histopathologically verified DKD; (3) age < 75 years; (4) availability of complete clinical data, including serum creatinine, urinary albumin, and estimated glomerular filtration rate (eGFR); and (5) absence of malignant tumors and other concomitant renal diseases based on clinical and pathological evaluation. Participants were excluded according to the following criteria: (1) concurrent malignant disease; (2) non-diabetic kidney disease or mixed renal pathology; (3) acute kidney injury at enrollment; or (4) incomplete clinical or pathological data. Patients were further stratified into early-stage and late-stage DKD according to renal function and albuminuria severity (Supplementary Tables S1 and S2). As the KDIGO classification does not explicitly define early- and late-stage DKD, biopsy-proven DKD was operationally categorized in this study based on estimated glomerular filtration rate (eGFR) and urinary albumin-to-creatinine ratio (UACR). Early-stage DKD included patients with eGFR ≥ 60 mL/min/1.73 m^2^ and UACR in categories A1 (< 30 mg/g) or A2 (30-300 mg/g), whereas late-stage DKD included those with eGFR < 60 mL/min/1.73 m^2^ and/or UACR in category A3 (> 300 mg/g).

### H&E staining, MT staining, IHC analysis, and immunofluorescence

Histological injury assessment utilized H&E staining, with glomerular and tubular damage scoring conducted as previously described^39^, ^40^. MT staining was used to assess interstitial fibrosis. IHC analysiswas performed as previously described^35^, ^36^, ^41^, utilizing these primary antibodies: anti-NAT10 (1:500), anti-FN (1:50 dilution), anti-a-SMA (1:100 dilution), anti-collagen IV (1:100 dilution), and anti-collagen I (1:100 dilution). Stained kidney sections were examined utilizing an Olympus microscope fitted with UV epi-fluorescence.

For immunofluorescence analysis, tissue sections were incubated overnight at 4 °C with primary antibody targeting NAT10 (1:500), then subjected to 1-hour incubation with fluorescent secondary antibody (Abcam, ab150116) at 37 °C under dark conditions. DAPI served for nuclear staining, and sections underwent visualization with a fluorescence microscope.

### Reverse transcription quantitative PCR (RT-qPCR)

Total RNA was isolated from kidney tissues or BUMPT cells employing Trizol reagent (Takara Bio Inc., Kusatsu, Japan) per the supplier's protocols. The extracted RNA underwent reverse transcription to generate first-strand cDNA utilizing the Prime Script RT Reagent Kit and gDNA Eraser (AG11711, Accurate Biotechnology (Hunan) Co., Ltd., Changsha, China) following established protocols^42^ ^43^. The resulting cDNA was used as the template for quantitative PCR, which was performed with SYBR Green dye (AG11701, Accurate Biotechnology, Hunan, China) on a LightCycler 96 system (Roche). The following primer pairs were utilized: *Nat10*: 5´-AAGACCACAACGACAGCCAGAC-3´ (forward) and 5´-TCCAGGCACAGCAGGTCATTAAG-3´ (reverse); *Brd4*: 5´-AAGATGCCTGATGAGCCTGAAGAG-3´ (forward) and 5´-ACTGTCGCTGTCGGAAGAACTG-3´ (reverse); *Pfkm*: 5´-GAATGCCGCTGTTCGCTCTACC-3´ (forward) and 5´-GCCAGACCCTCAAAGCCATCATG-3´ (reverse); *Actb*: 5´-GTGCTATGTTGCTCTAGACTTCG-3´ (forward) and 5´-ATGCCACAGGATTCCATACC-3´ (reverse). Relative quantification was performed using the 2-ΔΔCT methodology, whereas absolute quantification was executed based on a standard curve.

### Immunoblot analysis

Protein samples with comparable loading amounts were subjected to SDS-PAGE, followed by transfer onto nitrocellulose membranes (Amersham, Buckinghamshire, UK) as described previously^42^. After transfer, the membrane underwent washing with TBST buffer and blocking for 1 h using blocking buffer containing 5% milk. Subsequently, the blot underwent overnight incubation at 4 °C with primary antibodies targeting fibronectin (1:2,000 dilution), collagen I (1:1,000 dilution), a-SMA (1:1,000 dilution), NAT10 (1:1,000 dilution), and collagen IV (1:1,000 dilution), TGF-β1 (1:1,000 dilution), phospho-p65 (1:1,000 dilution), p65 (1:1,000 dilution), BRD4 (1:1,000 dilution), phospho-STAT3 (1:1,000 dilution), STAT3 (1:1,000 dilution), PFKM (1:1,500 dilution), Histone H3 (1:2,000 dilution), H3K18la (1:1,500 dilution), and β-Tubulin (1:2,000 dilution). After washing procedures, the membrane underwent incubation with secondary antibody (1:5,000). β-tubulin functioned as an internal loading control.

### Chromatin immunoprecipitation (ChIP) analysis

To verify NF-κB binding sites within *Nat10* promoter regions, a commercial ChIP assay kit (Millipore, Burlington, MA, USA) was employed. Treated cellular samples underwent ultrasonic fragmentation, then centrifugation for supernatant collection containing DNA fragments. These fragments underwent immunoprecipitation using phospho-p65-specific antibodies (Cat#3033S, Cell Signaling Technology, Danvers, MA, USA). The immunoprecipitated DNA was then analyzed and identified via RT-PCR. Precipitated DNA underwent RT-PCR analysis using specific primers:(F1R1): 5′-TGCCAGCACCTACACTTGTG-3′(forward) And 5′-ACACACGTAACACACCATCT-3′(reverse); (F2R2): 5′-GCCTGTGAGTTGCATACACCC-3′(forward) And 5′-CAAGGCTATGCAGGAAACTGCTC-3′(reverse); (F3R3): 5′-CCTAGATGGTGTGTTACGTGTGT-3′ (forward) and 5′-GCCCTACACTTTGGGTAGAAGCC-3′(reverse).

### Luciferase reporter assay

Luciferase reporter plasmids were constructed through cloning partial sequences from *Brd4* mRNA or *Pfkm* mRNA that contained either wild-type or mutated ac4C motifs (Corues Biotechnology Co., Ltd., Nanjing, Jiangsu, China). For luciferase activity analysis, BUMPT cells were plated in 24-well plates at 1 × 10⁵ cells per well and then transfected with plasmids using Lipofectamine 2000 (Invitrogen, PA530492, MA, USA). After 24 h post-transfection, luciferase activity was evaluated in harvested cells utilizing the Dual-Luciferase Reporter Assay System (GeneCreate, JKR23008, Wuhan, China). Renilla luciferase activity served to normalize the relative luciferase activity.

### N4-Acetylcytidine RNA immunoprecipitation sequencing (ac4C-RIP-seq) and ac4C-RIP-PCR

The ac4C-RIP-seq was conducted by CloudSeq Biotech Co., LTD (Shanghai, China). The ac4C-RIP procedure was performed using an RNA immunoprecipitation kit (Bes5101, Bersin Biotechnology, Guangzhou, China) per the supplier's protocols. In brief, cells underwent lysis in 1 mL of RIP lysis buffer for 20 min, and 100 μL lysate was preserved at -80 °C for subsequent analyses. The remaining lysate underwent incubation with anti-ac4C antibody (ab252215, Abcam, Cambridge, UK) or control mouse IgG (ab131368, Abcam, Cambridge, UK) under rotation at 4 °C for 16 h. Subsequently, antibody-bound RNA fragments were captured using Protein A/G magnetic beads through incubation at 4 °C for 1 h with continuous rotation. RNA was extracted utilizing the phenol-chloroform method. The purified RNA underwent RT-PCR analysis as previously described.

### RNA stability

To assess RNA stability, cells underwent transfection with either nonspecific control shRNA or *Nat10* shRNA, then received treatment with 5 µg/mL actinomycin D (Sigma, SBR00013) for transcription inhibition. Total RNA extraction from treated cells occurred at specified time intervals, with mRNA degradation analysis performed via RT-qPCR.

### Dot blot

RNA extraction from cellular samples was performed, with concentration normalization applied across all samples. A mixture comprising 20X SSC buffer and 37% formaldehyde at a 3:2 ratio was prepared, with equal RNA volumes subsequently added. The mixture underwent incubation at 95 °C for 5 min to achieve RNA denaturation. Subsequently, 2 μL of denatured RNA was spotted onto a nitrocellulose membrane and crosslinked under UV light at 302 nm. Membranes underwent quenching in phosphate-buffered saline with Tween-20 (PBST) and subsequent blocking with 5% skim milk for 2 h. Post-blocking, membranes received overnight incubation at 4 °C with an anti-ac4C antibody, followed by secondary antibody incubation at ambient conditions for 1 h the following day. Thorough PBST washing was conducted, and membranes received enhanced chemiluminescence reagent treatment. Visualization of ac4C levels was achieved using a chemiluminescence imaging system.

### Statistical analysis

Two-group comparisons were conducted with two-tailed Student's t-tests. For datasets containing more than two groups, either one-way or two-way ANOVA was performed as appropriate. Quantitative data are shown as mean ± SD, and statistical significance was defined as p < 0.05.

## Results

### NAT10 shows increased expression in renal biopsies from DKD patients and in DKD experimental models *in vitro* and *in vivo*

To examine NAT10 expression in DKD, urinary, serum, and renal biopsy specimens were first collected from patients with early-stage DKD (n = 13), late-stage DKD (n = 12), and kidney cancer (n = 9). Supplementary Table 1 provides a summary of the initial demographic and clinical data for all included patients. Among patients diagnosed with DKD, two distinct groups were established: early-stage and late-stage DKD, based on renal function deterioration (eGFR) and proteinuria levels, as shown in Supplementary Table 1. First, ELISA was performed to determine NAT10 levels in serum and urine samples from DKD patients, with findings demonstrating elevated NAT10 levels in both serum and urine from early-stage DKD patients, reaching peak levels in late-stage DKD patients (Figure 1A, B). Correlation analysis further showed that urinary NAT10 expression was positively linked to the urinary ACR and negatively linked to eGFR. Serum NAT10 expression was positively linked to serum creatinine (Figure 1C-E).

Furthermore, renal tissue specimens were obtained from patients with early- or late-stage DKD and from pathological controls (PC), with the latter comprising adjacent non-tumor renal tissues from patients with renal cell carcinoma (Supplementary Table 1). Histological evaluation and MT staining revealed increased tubular damage, glomerular enlargement, and interstitial fibrosis in kidney tissues from early-stage DKD patients compared to PC tissues, with these pathological changes becoming more pronounced in late-stage DKD (Figure 1F-I). Immunohistochemical analysis demonstrated that NAT10 and fibrotic markers, encompassing Collagen I, Collagen IV, and Fibronectin, were elevated in early-stage DKD tissues relative to PC tissues, with more marked upregulation observed in late-stage DKD (Figure 1F, J; Figure S1A-E). Correlation analysis demonstrated that the proportion of NAT10-positive cells was positively linked to tubular injury and the fibrotic area (Figure 1K, L). Subsequently, NAT10 expression was examined in renal tubular cells treated with high glucose and in DKD mouse kidneys induced by STZ. RT-qPCR analysis revealed that *Nat10* mRNA expression in BUMPT cells exhibited time-dependent elevation under high glucose (HG) treatment, while mannitol demonstrated negligible influence (Figure 1M). Consistently, both immunoblot and immunofluorescence staining analyses further confirmed the RT-qPCR findings (Figure 1N, O). Additionally, immunofluorescence staining further demonstrated that NAT10 exhibited nuclear localization (Figure 1P). Finally, diabetes was established in C57BL/6J mice through Streptozotocin (STZ) administration, with the sodium citrate (SC) group followed in parallel to 18 weeks. Relative to the SC group, STZ administration led to elevated blood glucose levels, urinary ACR, and kidney-to-body weight ratio at 8 weeks, with these alterations becoming more pronounced at 18 weeks post-treatment (Figure S1F-H). Histological evaluation and MT staining revealed mild tubular injury, glomerular hypertrophy, and interstitial fibrosis at 8 weeks, and these lesions became more severe at 18 weeks after STZ administration (Figure 1Q, and R-T). Furthermore, immunohistochemical staining demonstrated time-dependent induction of NAT10 expression in the kidneys of STZ-treated mice. Localization analysis confirmed that NAT10 was predominantly expressed in the nuclei of renal tubular cells (Figure 1Q, U). The mRNA level of *Nat10* was increased at 8 weeks and was further elevated at 18 weeks after STZ treatment (Figure 1V), which was also confirmed by immunoblot analysis (Figure 1W, X).

### NAT10 mediates HG-triggered renal fibrosis in BUMPT cells

Although NAT10 was upregulated following HG treatment, its role in renal fibrosis remained unclear. In this study, stable BUMPT cell lines with *Nat10* knockdown or overexpression were established through transfection with *Nat10*-shRNA or *Nat10* plasmids, respectively, followed by cellular exposure to normal glucose (NG) or HG conditions. Immunoblot analysis revealed that HG incubation elevated the expression levels of NAT10, FN, collagen I, and collagen IV. These increases were attenuated in cells with stable *Nat10* knockdown, whereas they were further enhanced in cells with stable *Nat10* overexpression (Figure 2A-D). These findings suggested that NAT10 served as a fibrosis promoter.

### NAT10 regulates mRNA stability of *Brd4* and *Pfkm* with ac4C modification

As previously established, NAT10 typically facilitates ac4C modification in mRNAs, thus promoting stability in downstream transcripts 18, 23. To further elucidate the mechanisms through which NAT10 promotes HG-induced fibrosis, three groups, including Scr-shRNA/NG, Scr-shRNA/HG, and *Nat10* shRNA/HG, were subjected to ac4C-RIP RNA-seq. Differentially expressed genes were presented in a heatmap (Figure 3A), and a fold change of ≥2 served as the cutoff threshold. The findings revealed that 1,737 genes were significantly upregulated in the Scr-shRNA/HG group versus the Scr-shRNA/NG group, whereas 986 genes were downregulated in the *Nat10* shRNA/HG group versus the Scr-shRNA/HG group. By overlapping the 1,737 upregulated genes with the 986 downregulated genes, 634 target genes regulated by *Nat10* under high-glucose incubation were identified (Figure 3B, supplementary Table S2). KEGG pathway enrichment analysis identified multiple significantly enriched pathways following *Nat10* knockdown under high-glucose conditions, including galactose metabolism and the cell cycle (Figure 3C). In the bubble plot, statistical significance is represented by the enrichment score [-log10(P value)], whereas the vertical order does not indicate statistical rank. These two pathways were prioritized because of their relevance to metabolic reprogramming, epigenetic regulation, and renal fibrosis.

Among genes that were upregulated by high glucose and downregulated following *Nat10* knockdown, *Pfkm* and* Brd4* exhibited the greatest fold-change magnitudes within the galactose metabolism and cell cycle pathways, respectively (Figure 3D), and were therefore selected for further validation. Subsequently, IGV analysis revealed prominent ac4C peaks corresponding to *Brd4* and *Pfkm*. As shown in Figure 3E, the ac4C peaks of *Brd4* and *Pfkm* were markedly increased in the Scr-shRNA/HG group versus the Scr-shRNA/NG group, but were reduced in the *Nat10* shRNA/HG group versus the Scr-shRNA/HG group. To determine the effect of NAT10 on *Brd4* and *Pfkm* expression, the mRNA levels of *Brd4* and *Pfkm* were first examined by qRT-PCR in *Nat10*-knockdown and control stable BUMPT cell lines treated with NG or HG. It was found that the HG-induced elevation in *Brd4* and *Pfkm* mRNA levels was significantly attenuated by *Nat10* knockdown (Figure 3F, G), and this result was further validated by immunoblot analysis (Figure 3H, I). Consistent with the ac4C-RIP-seq results, potential ac4C motifs within the modified CDS regions of *Brd4* mRNA (chr17: 32,432,500-32,432,700) and *Pfkm* mRNA (chr15: 98,022,906-98,022,940) were identified (Figure 3J). Thereafter, ac4C-RIP followed by RT-PCR showed significant enrichment of ac4C-modified *Brd4* and *Pfkm* mRNAs (Figure 3K, L).

Finally, the potential involvement of the predicted ac4C-modified regions in NAT10-mediated regulation of *Brd4* and *Pfkm* was evaluated using a luciferase reporter assay. The wild-type *Brd4* and *Pfkm* constructs contained the putative ac4C-modified regions, whereas the mutant constructs lacked these sites. *Nat10* overexpression markedly increased luciferase activity driven by *Brd4*-WT and *Pfkm* -WT, but not by the mutant constructs, in BUMPT cells (Figure 3M, N). To further determine whether NAT10 affects the mRNA stability of *Brd4* and *Pfkm*, stable Scr-shRNA and *Nat10*-shRNA BUMPT cell lines were treated with 5 μg/mL actinomycin-D, and cells were collected at the indicated time points for subsequent analysis. RT-qPCR findings demonstrated that *Nat10* knockdown markedly diminished the mRNA stability of *Brd4* and *Pfkm* at 6, 9, and 12 h (Figure 3O, P). Collectively, these findings indicated that NAT10 enhances the stability of *Brd4* and *Pfkm* mRNAs through ac4C modification.

### BRD4 mediates the pro-fibrotic effect of NAT10 via activation of the STAT3/TGF-β axis in BUMPT cells treated with HG

Previous studies have suggested that BRD4 serves a regulatory function in the STAT3/TGF-β pathway and promotes fibroblast activation 44. However, its function and underlying mechanism in renal tubular cells remain unclear. To confirm BRD4's function, BUMPT cells underwent transfection with *Brd4*-siRNA or Scr-siRNA for expression modulation. As expected, immunoblot analysis revealed that *Brd4* knockdown reduced the HG-induced expression of p-STAT3, TGF-β1, and fibrotic proteins, including FN, collagen I, and collagen IV, in BUMPT cells (Figure 4A, B). Subsequently, to determine whether BRD4 mediated the effect of NAT10, *Brd4* plasmids were transfected into stable *Nat10*-shRNA or Scr-shRNA BUMPT cell lines, followed by HG incubation. Immunoblot findings verified that *Brd4* overexpression reversed the impacts of *Nat10*-shRNA on STAT3/TGF-β pathway activation and fibrosis marker expression (Figure 4C, D). These observations suggested that NAT10 regulated the TGF-β pathway through the STAT3/TGF-β axis in BUMPT cells.

### PFKM mediates the lactate-producing effect of NAT10 in BUMPT cells with HG incubation

It was further investigated whether NAT10 promoted HG-induced lactate production through upregulation of PFKM, which is associated with lactate production (Figure 5A, B) 45, 46. To verify the role of PFKM, *Pfkm*-siRNA or Scr-siRNA was transfected into BUMPT cells. Immunoblot analysis confirmed that PFKM inhibition reduced the HG-induced production of pyruvate and lactate (Figure 5C-F). Subsequently, *Pfkm* plasmids were transfected into stable *Nat10*-shRNA or Scr-shRNA BUMPT cell lines. Consistently, the HG-induced increase in pyruvate and lactate secretion was suppressed by *Nat10* knockdown. Nevertheless, *Pfkm* overexpression reversed this effect (Figure 5G-J). Therefore, these findings indicated that NAT10 mediated HG-induced pyruvate and lactate secretion in BUMPT cells through upregulation of PFKM.

### NAT10 in BUMPT cells promotes the production of TGF-β1 and lactate and then activates the mouse kidney fibroblasts via the NOTCH1/SMAD3 pathway

Because renal tubular cells can stimulate interstitial fibroblasts through the secretion of profibrotic factors or lactate 21-25, it was investigated whether NAT10 in renal tubular cells could enhance profibrotic factor or lactate production and subsequently activate fibroblasts. Stable NAT10-overexpressing and control BUMPT cell lines underwent incubation with NG or HG for 48 h, after which the conditioned media were collected. To determine the contributions of TGF-β1 and lactate, TGF-β1 neutralizing antibody (Ab-TGF-β1) or NaHCO3 was added to the collected media, and NIH/3T3 fibroblasts were then cultured in the treated media for 24 h (Figure 6A). Conditioned medium obtained from BUMPT cells with NAT10-overexpression promoted Fibronectin, collagen I, and collagen IV expression in fibroblasts. This effect was markedly attenuated by TGF-β1 neutralizing antibody or NaHCO3, respectively. However, this inhibitory effect was further enhanced by the combined treatment with Ab- TGF-β1 and NaHCO3 (Figure 6B, C). To further examine the mechanism behind the synergistic effect of TGF-β1 and lactate, fibroblasts underwent stimulation with TGF-β1 with or without lactate presence. Lactate levels in the fibroblast indicated that TGF-β1 or lactate treatments may stimulate an increase of intracellular lactate, and this effect was enhanced by the combined treatment with TGF-β1 and lactate (Figure 6D). Immunoblot analysis demonstrated that lactate or TGF-β1 enhanced H3K18la, NOTCH1 and p-SMAD3 expression, along with Fibronectin, collagen I, and collagen IV expression. These effects were further strengthened by the combined treatment with TGF-β1 and lactate (Figure 6E, F). To further clarify how lactate promoted the expression of NOTCH1 and p-SMAD3, H3K18la mutant plasmids or H3K18la overexpression plasmids were transfected into NIH/3T3 fibroblasts. Immunoblot results demonstrated that lactate might regulate the expression of NOTCH1 and p-SMAD3 through H3K18 lactylation (Figure 6G, H). RT-qPCR analysis showed that lactate treatment significantly increased *Notch1* mRNA expression in NIH/3T3 cells. H3K18la overexpression further enhanced this increase, whereas the H3K18R mutant attenuated lactate-induced *Notch1* expression (Figure 6I). A potential H3K18la-enriched region was identified within the *Notch1* promoter (Figure 6J). ChIP-PCR analysis further demonstrated that lactate treatment increased H3K18la enrichment at this promoter region (Figure 6K). These findings suggest that lactate-induced H3K18 lactylation promotes *Notch1* transcription by increasing H3K18la enrichment at the *Notch1* promoter.

### Upregulation of NAT10 during HG incubation is directly regulated by NF-κB and further enhanced by lactate

As previously documented, NF-κB becomes activated within tubular epithelial cells during DKD 47. Therefore, it was hypothesized that NF-κB might serve as an upstream regulator of NAT10 during HG treatment. Immunoblot results showed that HG treatment induced NAT10 expression and NF-κB p65 activation at 24 h, with both reaching peak levels at 48 h (Figure 7A, B). The impact of BAY 11-7082, a frequently utilized NF-κB signaling inhibitor, was subsequently evaluated. Immunoblot analysis revealed that BAY 11-7082 markedly diminished HG-induced NAT10 expression and NF-κB p65 activation (Figure 7C, D). To investigate how NF-κB regulates NAT10 expression, three potential NF-κB-binding sites within the *Nat10* promoter were predicted utilizing the JASPAR database (https://jaspar.elixir.no/) (Figure 7E). ChIP analysis further showed that HG promoted p-p65/NF-κB occupancy at these binding sites (Figure 7F). Subsequently, luciferase reporter plasmids containing WT *Nat10* promoter sequences or different mutations at the predicted binding sites were separately co-transfected with an NF-κB overexpression plasmid into BUMPT cells. Luciferase activity assays revealed that promoter activity was markedly elevated in the NAT10 WT group following NF-κB overexpression (Figure 7G). In contrast, luciferase activity was reduced in the mutation1, mutation2, and mutation3 groups. Based on the preceding findings, it was further hypothesized that lactate-induced lactylation might contribute to NF-κB-mediated NAT10 expression. Lactate treatment induced dose- and time-dependent increases in H3K18la, p-p65, and NAT10 protein levels, whereas total p65 remained relatively unchanged (Figure 7H, I, K, L). RT-qPCR analysis further showed that Nat10 mRNA expression increased with both lactate concentration and exposure time (Figure 7J, M). Together, these results indicate that lactate promotes NAT10 expression in association with enhanced H3K18 lactylation and NF-κB activation. H3K18la mutant plasmids, in which the amino acid site at H3K18 was mutated to inhibit lactylation, or H3K18la overexpression plasmids, were then transfected into BUMPT cells. The results demonstrated that alteration of the H3K18la lactylation level positively affected p-p65 activation and NAT10 expression under lactate treatment (Figure 7N, O). Finally, it was investigated whether H3K18la directly regulated NAT10 expression or acted through p-p65/NF-κB activation. H3K18la overexpression markedly enhanced lactate-induced p-p65 activation and NAT10 expression, whereas this effect was substantially blocked by BAY 11-7082 treatment (Figure 7P, Q). These results demonstrated that lactate promoted NAT10 expression by activating p65/NF-κB signaling in an H3K18la-dependent manner. Collectively, these findings indicated that HG directly activated p65/NF-κB to induce NAT10 expression, while HG-induced lactate further amplified this effect, thereby forming a positive feedback loop.

### NAT10 deletion in tubular cells mitigates renal fibrosis in the STZ-induced DKD model via inhibiting BRD4/STAT3/TGF-β1 and PFKM/lactate/H3K18la axes

To confirm the *in vitro* findings, a kidney tubule-specific NAT10-knockout (T-NAT10-KO) mouse model was generated. The breeding protocol is shown in Supplementary Figure 2A. The genotypes of T-NAT10-KO mice were confirmed by PCR analysis (Supplementary Figure 2B). Immunoblot analyses verified that renal NAT10 expression was lower in T-NAT10-KO mice than in T-NAT10-WT mice under SC buffer and STZ treatment conditions (Supplementary Figure 2C, D). Age-matched male littermate T-NAT10-WT and T-NAT10-KO mice were randomly assigned to SC and STZ groups. At 18 weeks after STZ administration, T-NAT10-WT mice exhibited increases in kidney-to-body weight ratio (KW/BW), urinary albumin/creatinine ratio (ACR), serum creatinine, and BUN levels. Tubule-specific *Nat10* knockout markedly alleviated these renal functional abnormalities without altering the degree of hyperglycemia (Supplementary Figure 3A-E). H&E and MT staining demonstrated that STZ induced tubular injury and renal fibrosis in T-NAT10-WT mice, and these effects were attenuated by T-NAT10-KO (Figure 8A-C). Immunohistochemical analyses further confirmed that T-NAT10-KO reduced the STZ-induced expression of fibrotic proteins, encompassing Collagen I, Collagen IV, Fibronectin, and α-SMA (Figure 8D-H). Immunoblot analysis showed that T-NAT10-KO decreased the STZ-induced expression of NAT10, fibrotic markers, BRD4, STAT3, and TGF-β1 (Figure 8I-L). Immunoblot and lactate assays additionally demonstrated that T-NAT10-KO inhibited PFKM and H3K18la expression and reduced lactate production in STZ-induced diabetic kidneys (Figure 8K-M). These data indicated that T-NAT10-KO mitigated STZ-induced renal fibrosis by inhibiting the BRD4/STAT3/TGF-β1 and PFKM/lactate/H3K18la axes.

### Pharmacological inhibiting of NAT10 potently suppresses HG-induced renal fibrosis in BUMPT cells

Inhibition of target gene expression with small-molecule compounds represents a promising therapeutic approach for disease intervention. To identify novel pharmacological inhibitors of NAT10 and explore potential candidates for DKD treatment, 200 small-molecule compounds were initially evaluated by in silico virtual screening and molecular docking. Subsequently, seven candidate compounds were selected from stable NAT10-overexpressing cell lines on the basis of fluorescence intensity analysis (Figure 9A, Supplementary Figure S4A-C). Finally, GDP disodium salt (HY-113066A) was identified by immunoblot analysis as the compound exhibiting the strongest inhibitory effect. GDP disodium salt was selected as a potential therapeutic agent for anemia of inflammation based on its reported ability to disrupt the hepcidin-ferroportin axis and modulate IL-6/STAT3 signaling 48. In addition, as a purine nucleoside diphosphate, it functions as a stimulator of ATP-sensitive K⁺ channels 49.Molecular docking analysis revealed that GDP disodium salt interacted with the Ac-CoA-binding pocket of NAT10, with a docking score of -8.435 (Figure 9B). Treatment of BUMPT cells with GDP disodium salt or the NAT10 inhibitor remodelin significantly reduced cell proliferation, with half-maximal inhibitory concentrations (IC_50_) of 66.48 μM and 123.7 μM, respectively (Figure 9C). Therefore, cellular RNA ac4C levels were reduced more efficiently by GDP disodium salt than by remodelin (Figure 9D). Likewise, GDP disodium salt reduced NAT10 protein levels in a dose- and time-dependent manner in stable *Nat10*-overexpressing BUMPT cell lines (Figure 9E-H), whereas it had no significant effect on *Nat10* mRNA levels (Figure S4E). Immunoblot analysis showed that GDP disodium salt suppressed the HG-induced expression of NAT10, fibrotic markers, BRD4, STAT3, TGF-β1, PFKM, and H3K18la (Figure 9I-L). Lactate levels were also reduced by GDP disodium salt (Figure 9M). To further clarify the mechanism by which GDP disodium salt inhibited NAT10, stable *Nat10*-overexpressing BUMPT cell lines were treated with GDP disodium salt, with or without MG132. The results confirmed that GDP disodium salt suppressed NAT10 expression, and this effect was partially counteracted by the ubiquitination inhibitor MG132 (Figure 9N, O). Furthermore, ubiquitination assays showed that GDP disodium salt significantly increased NAT10 ubiquitination in the presence of MG132 (Figure 9P). Notably, transient transfection of a *Nat10* overexpression plasmid partially rescued the effects of GDP disodium salt (Figure 9Q-T). These data suggested that GDP disodium salt reduced NAT10 expression by increasing NAT10 ubiquitination.

### GDP disodium salt alleviates diabetes-induced renal fibrosis in *db/db* mice through NAT10 inhibition

To determine the optimal dose and administration interval for intraperitoneal injection, C57BL/6 mice were intraperitoneally injected with GDP disodium salt at doses of 2.5, 5, and 10 mg/kg, respectively. NAT10 expression was assessed 48 h after injection, and immunoblot analysis showed that GDP disodium salt at 10 mg/kg exerted the strongest inhibitory effect. Subsequently, mice received intraperitoneal injections of GDP disodium salt at an optimal dose of 10 mg/kg, with NAT10 expression assessed at 24, 48, and 72 h following administration. Immunoblot results demonstrated that the inhibitory effect on NAT10 was most pronounced at 72 h (Figure 10A-D). Thereafter, 8-week-old *db/db* and *db/m* mice were intraperitoneally injected with GDP disodium salt at 10 mg/kg or PBS as a control once every 3 days until 18 weeks of age. Functionally, 10 weeks of GDP disodium salt administration decreased the kidney-to-body weight ratio, urinary ACR, serum creatinine, and BUN levels in *db/db* diabetic mice while maintaining unchanged blood glucose levels (Supplementary Figure S5A-E). Histological examination and MT staining revealed that GDP disodium salt treatment markedly alleviated glomerular and tubular injury, as well as renal fibrosis (Figure 10E-H). To preliminarily assess its in vivo hepatic safety, serum ALT and AST levels were measured. GDP disodium salt treatment did not significantly alter either ALT or AST levels compared with PBS-treated *db/db* mice, indicating no detectable hepatic injury under the present treatment conditions (Supplementary Figure S5F-G). Immunohistochemical and immunoblot analyses further showed that GDP disodium salt treatment suppressed NAT10 expression and fibrotic proteins, including Collagen I, Collagen IV, Fibronectin, and α-SMA, in the kidneys of *db/db* mice (Figure 10I-M). Immunoblot analysis further demonstrated that GDP disodium salt treatment inhibited NAT10 expression along with the expression of its downstream genes BRD4, p-STAT3, and TGF-β1 in the kidneys of *db/db* mice (Figure 10N-Q). Meanwhile, immunoblot and lactate detection assays showed that GDP disodium salt inhibited PFKM expression and reduced lactate levels and H3K18 lactylation (Figure 10P-R). These results suggested that GDP disodium salt may protect the kidney against DKD by inhibiting NAT10.

## Discussion

DKD is distinguished by pathological characteristics that differ from those observed in other forms of CKD 50-54. Although TIF is a shared pathological hallmark of CKD, it is primarily observed during the advanced stages of DKD 50, 51, 55. In this investigation, NAT10 demonstrated upregulation by NF-κB within renal tubular cells following HG treatment. Furthermore, lactate generated in tubular cells in response to HG was shown to promote H3K18la expression, thereby enhancing NF-κB activation and subsequently inducing NAT10 expression, ultimately establishing a positive feedback loop. Suppression of NAT10 attenuated HG-induced renal fibrosis. Mechanistically, NAT10 in tubular cells promoted the expression of BRD4 and PFKM, thereby activating the STAT3/TGF-β1 axis and increasing lactate production; both TGF-β1 and lactate jointly activated fibroblasts through the H3K18la/NOTCH/SMAD3 axis (Figures 4, 5, 6). Notably, renal tubule-specific *Nat10* deletion and GDP disodium salt treatment both ameliorated TIF in DKD mice (Figure 8 and 10). Collectively, these results reveal previously unrecognized regulatory events contributing to renal fibrotic progression in DKD, broaden the current understanding of DKD pathogenesis, and identify a potential therapeutic compound for preventing DKD progression through pharmacological inhibition of NAT10.

NAT10, a 982-amino acid nucleocytoplasmic protein, was initially identified as aberrantly expressed in colorectal cancer and as being associated with tumor invasion 56. However, an increasing volume of recent studies has shown that NAT10 participates in various diseases, including gastric cancer, obesity, cardiac ischemia-reperfusion (I/R) injury, renal cell carcinoma, and acute kidney injury (AKI) 57-60. It has been demonstrated in two studies that NAT10 promotes myocardial fibrosis following myocardial infarction 29, 31. More recent evidence has further shown that NAT10 accelerates the progression of pulmonary fibrosis through acetylation of TGF-β1 32. Moreover, NAT10-mediated ac4C modification of TGF-β1 mRNA has been shown to drive cellular senescence and hepatic fibrosis 30. However, the function of NAT10 in DKD pathogenesis and renal fibrosis remains poorly characterized. This study represents the initial demonstration that tubular cell NAT10 mediates renal fibrosis progression both *in vitro* and *in vivo* (Figures 1, 2). Additionally, ac4C-RIP-seq profiling revealed that NAT10 is associated with a broad range of gene transcripts in cells under HG or diabetic conditions. Subsequent validation experiments, including ac4C-RIP assays and luciferase reporter assays, showed that *Brd4* and *Pfkm* mRNAs were enriched for ac4C modification and that the predicted ac4C-modified CDS regions contributed to NAT10-mediated regulation of their mRNA stability (Figure 3).

BRD4 functions as an epigenetic reader that binds acetylated lysine residues on histones and promotes the formation of transcriptional regulatory complexes^61^-^63^. Notably, this factor has also been widely associated with the development of fibrosis in the liver, lung, and atrium^64^-^66^. Previous investigations have suggested that BRD4 mediates TGF-β1-induced renal fibrosis in unilateral ureteral obstruction surgery and the adenosine model 67. Another study reported that TGF-β1 contributes to diabetic nephropathy progression through regulation of BRD4 and its downstream NOTCH1/YAP signaling axis 68. TGF-β has also been reported to regulate BRD4-associated STAT3 signaling during keloid progression 44. Additionally, the expression of TGF-β may be directly promoted by STAT3 in T cells 69. Collectively, these studies indicate the existence of a mutual regulatory interaction between BRD4 and TGF-β1 signaling via direct binding with STAT3 proteins. In this investigation, it was demonstrated that NAT10 in tubular cells promotes BRD4 expression and subsequently activates STAT3 to induce TGF-β1 production, thereby driving fibroblast activation through the NOTCH1/SMAD3 signaling pathway (Figure 4,6). Notably, tubule-specific deletion or pharmacological inhibition of NAT10 in tubular cells suppressed BRD4 expression, p-STAT3 activation, TGF-β1 expression, and renal interstitial fibrosis in db/db and STZ-induced diabetic mice (Figures 8 and 10).

Accumulating evidence supports a close association between fibrosis and metabolic reprogramming marked by enhanced glycolysis 70-72. Aerobic glycolysis refers to a metabolic state in which energy production mainly depends on glycolysis, while mitochondrial oxidative phosphorylation contributes to a lesser extent. This metabolic phenotype is marked by increased expression of enzymes involved in glycolysis and elevated levels of glycolysis-derived metabolites, especially lactate. In type 2 diabetic patients, elevated lactate dehydrogenase A (LDHA) expression, a critical glycolytic enzyme, is linked to a 45% higher DKD risk 73. Another investigation demonstrated that elevated urinary lactate metabolite concentrations are linked to renal fibrosis among DKD patients 74. LDHA levels were elevated in renal tubular epithelial cells, and renal lactate levels were markedly heightened in 12-week-old *db/db* mice 75. A strong positive correlation was identified between renal lactate concentrations and the expression of renal pro-fibrotic factors 75. Previous work showed that activation of glycolytic metabolism in renal tubular epithelial cells drives epithelial-mesenchymal transition and worsens renal fibrotic injury^76^. In addition, G protein-coupled receptors have been reported to contribute to renal fibrosis by enhancing glycolytic activity and inducing mitochondrial damage^77^. Although glycolytic metabolism has been implicated in renal fibrosis, how this metabolic process contributes to fibrotic progression remains incompletely understood. Therefore, this study validates an important association between altered glucose metabolism and renal fibrotic injury, and indicate an unrecognized profibrotic function of glycolysis in DKD (Figure 5; Figure 8; Figure 10).

As a product of glycolytic metabolism, lactate contributes to renal fibrotic progression by providing lactyl groups for histone lysine lactylation, thereby modifying protein activity and transcriptional regulation^78^-^81^. Histone lactylation regulates renal fibrosis 82. Recent investigations have demonstrated that lactate stimulates the KLF5 signaling pathway via histone H3K14 lactylation, thereby promoting epithelial-mesenchymal transition during the pathogenesis of DKD 75. PFKFB3, an enzyme involved in glycolysis, has been reported to facilitate renal fibrosis by enhancing histone lactylation, which in turn activates NF-κB signaling^82^. Histone lactylation has been shown to accelerate fibrosis in clear cell renal cell carcinoma by promoting the proliferative activity of renal tumor-associated fibroblasts^83^. Nevertheless, the detailed mechanism by which histone lactylation participates in renal fibrosis during DKD remains to be clarified. In this study, PFKM, a key regulator of glycolysis, was shown to be markedly upregulated under high-glucose conditions. NAT10 in tubular cells promoted PFKM expression and increased lactate production, which in turn increased lactate levels, enhanced histone H3K18 lactylation in fibroblast and promoted fibroblast activation through the NOTCH1/SMAD3 signaling pathway (Figure 5; Figure 6). This effect was further enhanced by TGF-β.

GDP disodium salt, classified as a purine nucleoside diphosphate, has been reported to activate ATP-sensitive potassium channels. It has been utilized to examine the kinetics and properties of GTPases^49^. GDP disodium salt has been reported as a potential therapeutic candidate for anemia of inflammation through regulation of the hepcidin-ferroportin axis and IL-6/STAT3 signaling^48^. In this study, GDP disodium salt was found to suppress NAT10 protein expression by enhancing NAT10 ubiquitination, with greater efficacy than remodelin, a recognized inhibitor. However, the following limitations should be acknowledged: 1) the binding affinity and binding sites of GDP disodium salt for NAT10 need to be further explored; 2) the specific mechanism through which GDP disodium salt promotes NAT10 protein degradation remains to be investigated 3) Although GDP disodium salt did not significantly affect serum ALT or AST levels under the present experimental conditions, systemic inflammatory markers were not assessed. Further studies are therefore required to evaluate its potential systemic inflammatory effects.

Taken together, our findings provide the first evidence that NAT10 in tubular cells contributes to TIF during DKD progression. Mechanistically, NAT10 induced by NF-κB was shown to stabilize *Brd4* and *Pfkm* mRNA through ac4C modification, thereby activating the STAT3/TGF-β and lactate/H3K18 lactylation pathways, which synergistically drive fibroblast activation through the NOTCH/SMAD3 axis. Additionally, lactate was also found to activate NF-κB, thereby promoting NAT10 expression and downstream pathway activation via H3K18 lactylation, ultimately forming a positive feedback loop. Finally, it was demonstrated for the first time that intervention with GDP disodium salt significantly attenuated renal functional impairment and fibrosis in diabetic *db/db* mice. Collectively, these findings support a pro-fibrotic role for NAT10, provide new mechanistic insight into DKD progression, and suggest GDP disodium salt as a potential therapeutic candidate for DKD.

## Supplementary Material

Supplementary figures and tables.

## Figures and Tables

**Figure 1:**
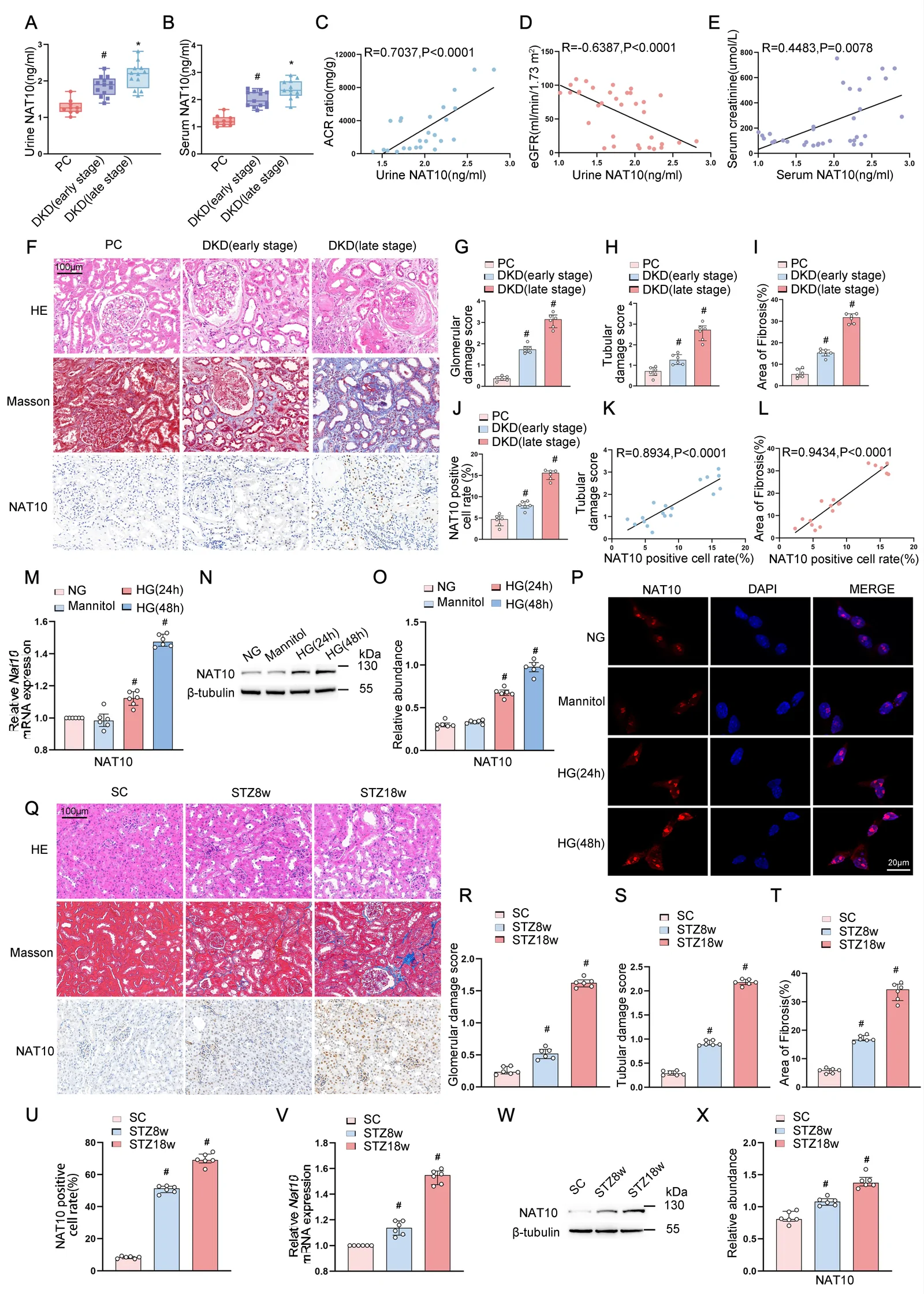
** NAT10 expression is elevated in renal biopsies from DKD patients and in DKD models both in vitro and in vivo.** (A, B) Urinary and serum NAT10 concentrations were determined by ELISA in patients with renal cell carcinoma(n=9), early-stage DKD(n=13), and late-stage DKD(n=12). # P< 0.05, versus pathological control (PC) group, * P < 0.05, versus DKD early-stage group. (C, D) Associations between urinary NAT10 expression and clinical parameters, including the urinary ACR and eGFR, were examined. (E) Correlations between serum NAT10 levels with serum creatinine. (F-L) Renal pathological changes and NAT10 expression in renal biopsy specimens from patients with DKD (early and late stage) and adjacent non-tumor renal tissues from the PC group. (F) Representative H&E staining, Masson staining, and immunohistochemical staining for NAT10 in human kidney samples. Scale bars, 100 μm.(G) Glomerular damage was assessed quantitatively. # P < 0.05, versus PC group. (H) Tubular damage was assessed quantitatively. # P < 0.05, versus PC group. (I) TIF was measured. # P < 0.05, versus PC group. (J) Assessment of NAT10-positive cell percentages by immunohistochemical staining. # P < 0.05, versus PC group. (K) Association analysis of NAT10-positive cell percentages with tubular damage. (L) Association analysis of NAT10-positive cell percentages with fibrotic area. (M-P) BUMPT cells were exposed to various treatments, including NG (5 mmol L⁻¹ D-glucose), HG (30 mmol L⁻¹ D-glucose), or mannitol (osmotic control, consisting of 5 mmol L⁻¹ D-glucose and 25 mmol L⁻¹ D-mannitol), for 24-48 h.(M) *Nat10* mRNA expression was measured using RT-qPCR. # P < 0.05, versus NG group. (N, O) NAT10 protein expression was assessed by immunoblot, followed by densitometric analysis. # P < 0.05, versus NG group. (P) Immunofluorescence staining was performed to assess NAT10 expression and subcellular localization within BUMPT cells. Scale bars, 20 μm. (Q-X) C57BL/6J mice received intraperitoneal streptozotocin (STZ) administration at 50 mg kg⁻¹ body weight daily for five consecutive days to induce diabetes, while control mice received sodium citrate (SC). Mice with fasting blood glucose concentrations exceeding 200 mg dL⁻¹ in two consecutive measurements were considered diabetic. Diabetic mice were assessed at 8 and 18 weeks, and SC mice at 18 weeks. (Q) Representative images of H&E, Masson, and immunohistochemical staining for NAT10 in kidney tissues. (R) Glomerular damage was assessed quantitatively. #P < 0.05, versus SC group. Scale bars, 100 μm.(S) Tubular damage was assessed quantitatively. # P < 0.05, versus SC group. (T) TIF was measured. # P < 0.05, versus SC group. (U) Assessment of NAT10 expression in renal tissues was conducted via immunohistochemical staining quantification. # P < 0.05, versus SC group. (V) *Nat10* mRNA concentrations in diabetic kidney tissues were determined using RT-qPCR. # P < 0.05, versus SC group. (W, X) Protein expression of NAT10 in diabetic kidney samples was analyzed by immunoblotting, with subsequent densitometric quantification. # P < 0.05, versus SC group. All other quantitative data are presented as mean ± SD (n = 6).

**Figure 2:**
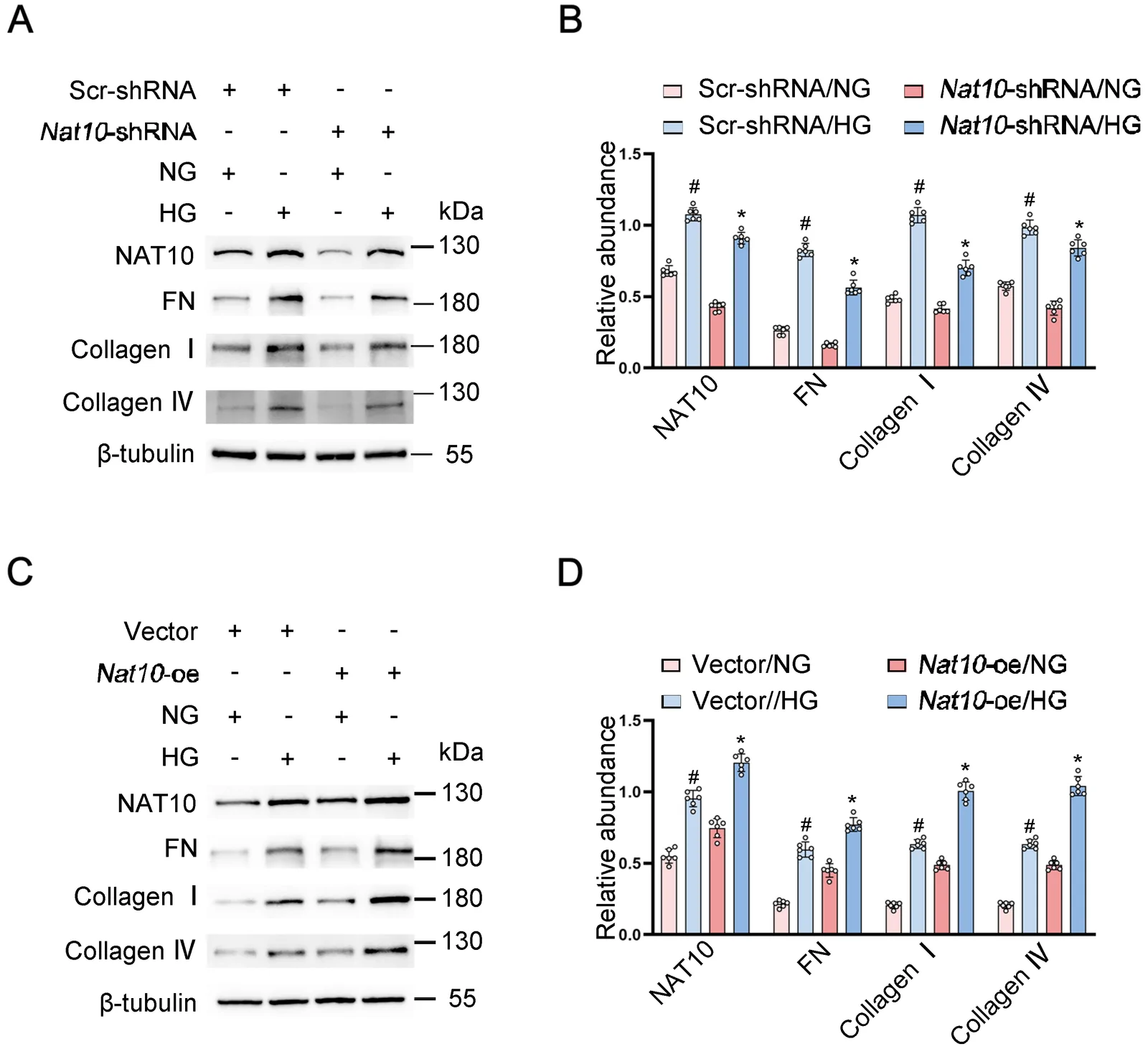
** NAT10 promotes fibrotic protein expression in BUMPT cells under high-glucose conditions.** (A) BUMPT cells were transfected with *Nat10* shRNA or scrambled shRNA (Scr-shRNA) and were then cultured under either HG or NG conditions. Cell lysates were subjected to immunoblot analysis of Collagen I, Collagen IV, Fibronectin (FN), NAT10, and β-tubulin. (B) Immunoblot bands were assessed by densitometric analysis. # P < 0.05, versus Scr-shRNA/NG group. * P < 0.05, versus Scr-shRNA /HG group. (C) Stable *Nat10*-overexpressing (*Nat10*-oe) BUMPT cell lines were cultured in NG or HG for 48 h, followed by immunoblot analysis of lysates to detect Collagen I, Collagen IV, FN, NAT10, and β-tubulin. (D) Immunoblot bands were assessed by densitometric analysis. # P < 0.05, versus Vector/NG group. * P < 0.05, versus Vector/HG group. Data are presented as mean ± SD (n = 6).

**Figure 3:**
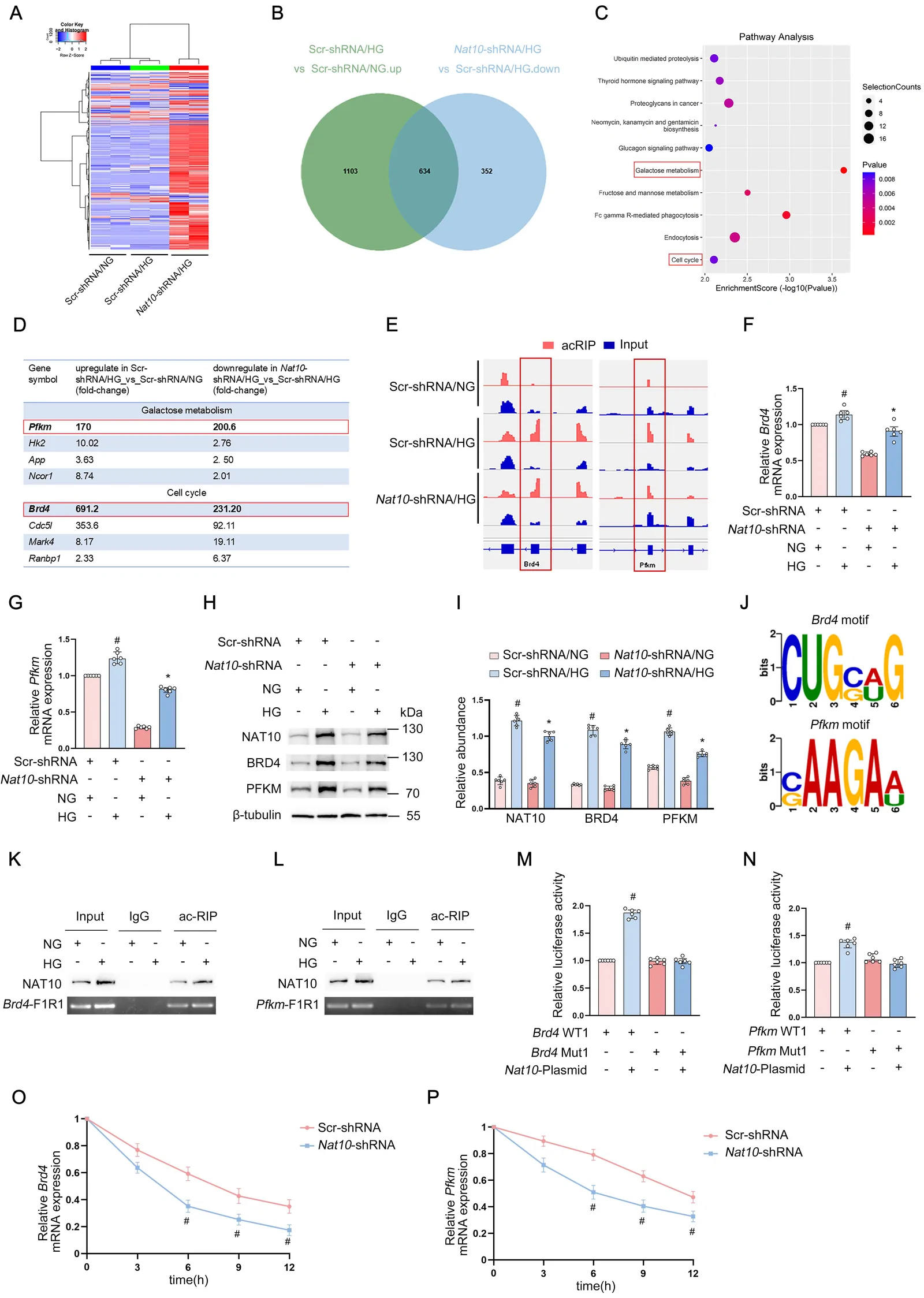
** NAT10 regulates mRNA stability of *Brd4* and *Pfkm* through ac4C modification.** ac4C-RIP-seq analysis was performed on three groups of BUMPT cells (Scr-shRNA/NG, Scr-shRNA/HG, and *Nat10*-shRNA/HG) to examine NAT10's influence on mRNA ac4C modification (n = 2). (A) Heatmap displaying differential ac4C-modified transcripts. (B) Venn diagram shows genes upregulated in the Scr-shRNA/HG group versus the Scr-shRNA/NG group and downregulated in the *Nat10* shRNA/HG group versus the Scr-shRNA/HG group. (C) KEGG Pathway Enrichment Analysis. The x-axis represents the enrichment score, defined as -log10(P value); bubble color represents the corresponding P value, and bubble size represents the number of differentially expressed genes assigned to each pathway. The vertical order of the pathways does not indicate statistical rank. (D) Differentially expressed genes associated with galactose metabolism and cell cycle (fold change > 2). (E) Integrative Genomics Viewer (IGV) tracks displayed ac4C-seq read distribution patterns within *Brd4* and *Pfkm* mRNA transcripts in BUMPT cells. Boxes indicate reduced ac4C peaks in *Nat10*-shRNA/HG group relative to Scr-shRNA/HG group. (F-I) Scr-shRNA and *Nat10*-shRNA cell lines were incubated under HG or NG conditions. (F, G) RT-qPCR analysis for *Brd4* and *Pfkm*. (H) Immunoblot analysis of NAT10, BRD4, and PFKM proteins in cell lysates. (I) Immunoblot bands were assessed by densitometric analysis. # P < 0.05, versus Scr-shRNA/NG group. * P < 0.05, versus Scr-shRNA /HG group. (J) Motif analysis of *Brd4* and *Pfkm* ac4C peaks within ac4C peaks in ac4C-RIP seq. (K, L) ac4C-RIP-PCR of *Brd4* and *Pfkm* predicted binding sites with the highest score. (M, N) Luciferase reporter assays using wild-type or mutant constructs. #P < 0.05 versus *Brd4* WT group or *Pfkm* WT group. (O, P) Stable BUMPT cells expressing Scr-shRNA or *Nat10*-shRNA were treated with actinomycin D (5 μg/mL), and *Brd4* and *Pfkm* mRNA levels were measured by RT-qPCR at the indicated time points. # P < 0.05 versus Scr-shRNA group. All other quantitative data are presented as mean ± SD (n = 6).

**Figure 4:**
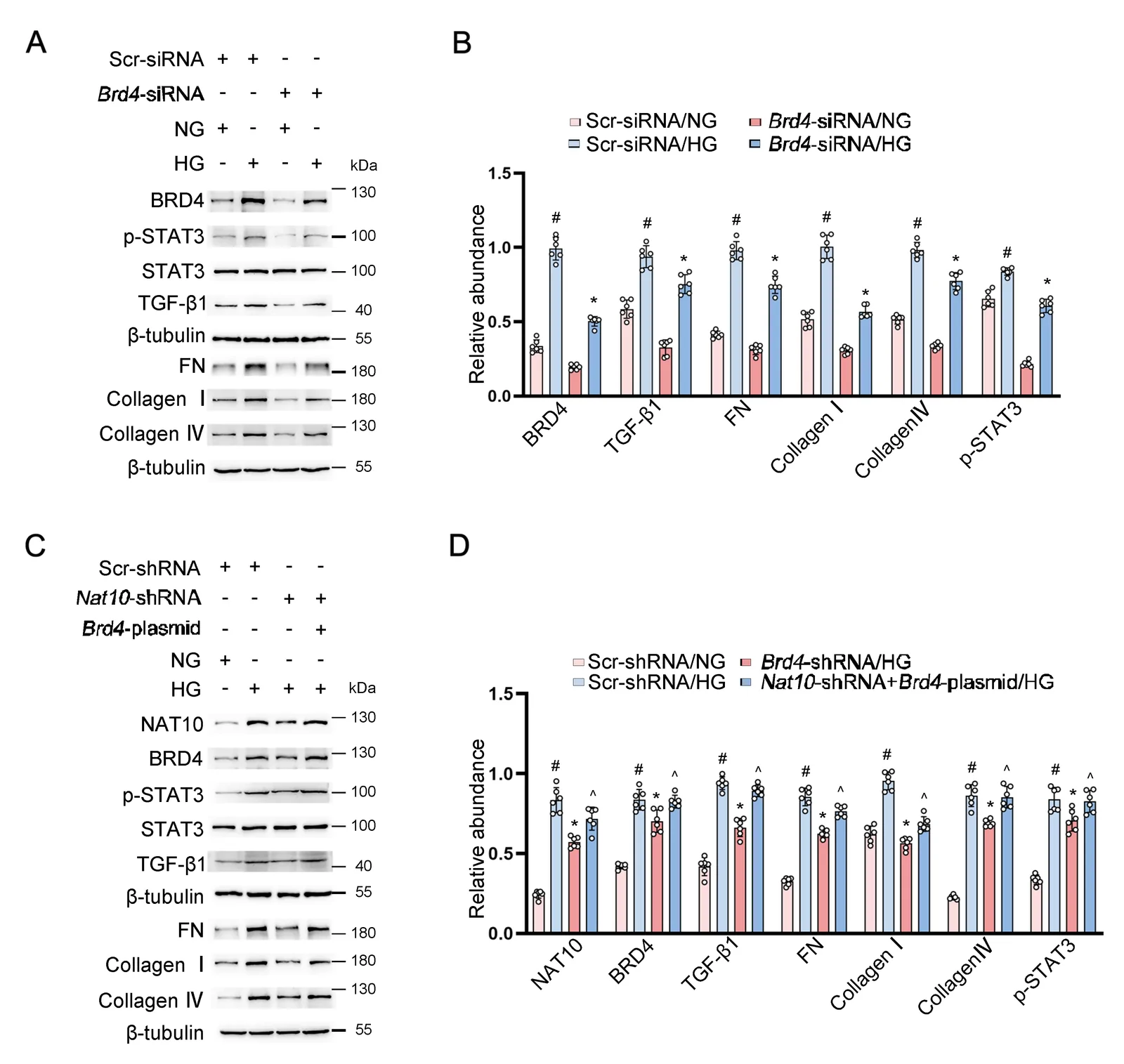
** BRD4 mediates NAT10's profibrotic effects through activation of the STAT3/TGF-β axis in BUMPT cells with HG-incubation.** (A) BUMPT cells underwent transfection with Scr-siRNA or *Brd4*-siRNA and were exposed to HG for 48 h. Immunoblot analysis was conducted to assess protein expression, with representative blots presented. (B) Immunoblot bands were assessed by densitometric analysis. # P < 0.05 versus Scr-siRNA/NG group. * P < 0.05, versus Scr-siRNA /HG group. (C) Stable BUMPT cells expressing Scr-shRNA or *Nat10*-shRNA were transfected with either an empty vector or a *Brd4* overexpression plasmid and then exposed to HG for 48 h. Cell lysates were subjected to immunoblot analysis of the indicated proteins, with representative blots displayed. (D) Immunoblot bands were assessed by densitometric analysis. # P < 0.05 versus Scr-shRNA/NG group. * P < 0.05, versus Scr-shRNA /HG group. ^ P < 0.05, versus *Nat10*-shRNA /HG group. Data are presented as mean ± SD (n = 6).

**Figure 5:**
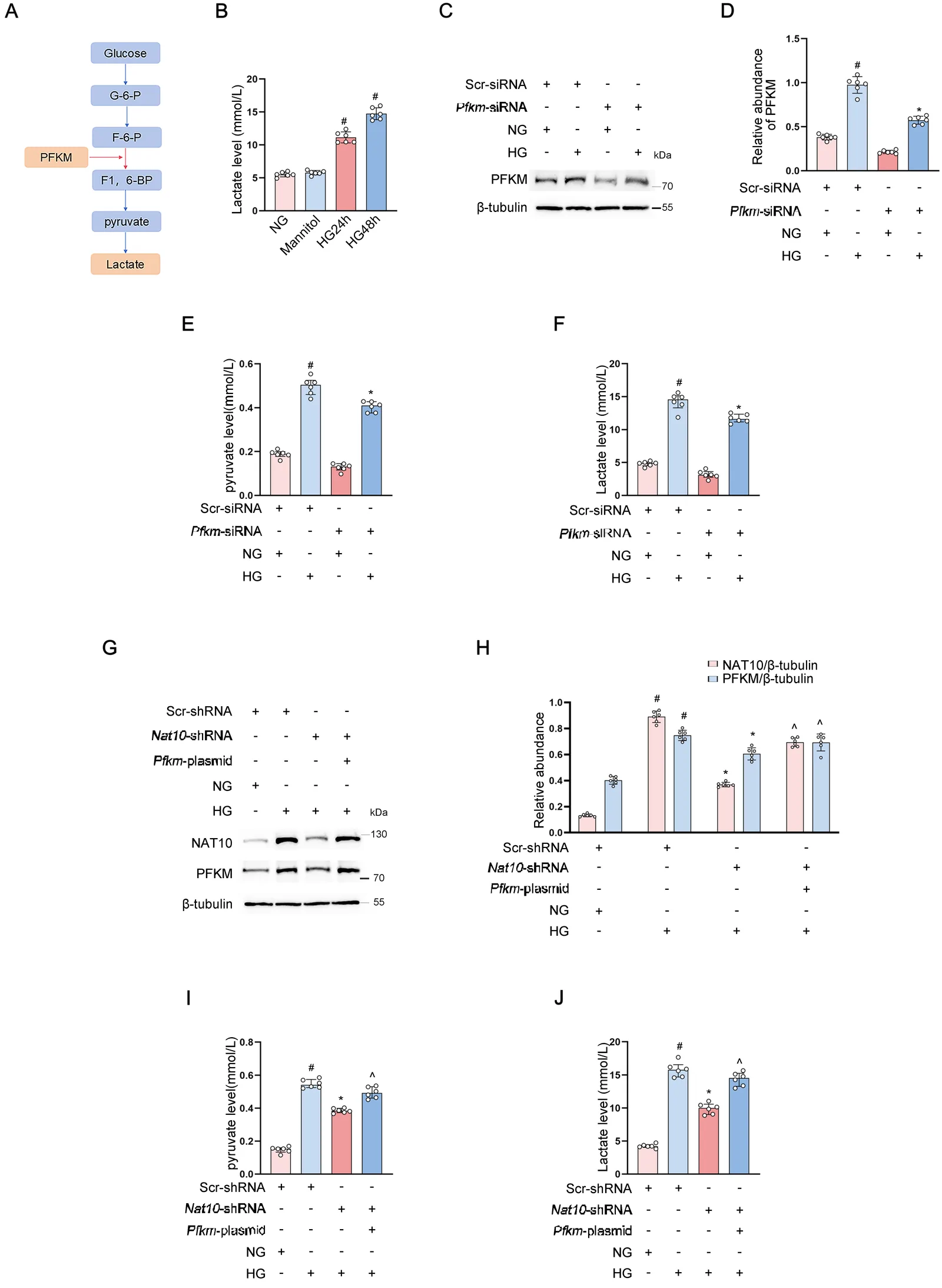
** PFKM regulates pyruvate and lactate production in BUMPT cells under high-glucose conditions.** (A) A schematic diagram illustrating role of PFKM in glycolysis and lactate production. (B) Lactate concentrations at NG, M, and HG at 24 h and 48 h in BUMPT cells. # P < 0.05 versus NG group. (C) BUMPT cells transfected with Scr-siRNA or *Pfkm*-siRNA were exposed to HG for 48 h, followed by immunoblot analysis of lysates for the specified proteins. Representative blots are shown. (D) Immunoblot bands were assessed by densitometric analysis. (E) Relative pyruvate levels in BUMPT cells following Scr/*Pfkm*-siRNA transfection and subsequent HG treatment. (F) Relative lactate levels in BUMPT cells after Scr-siRNA or *Pfkm*-siRNA transfection. # P < 0.05 versus Scr-siRNA/NG group. * P < 0.05, versus Scr-siRNA /HG group. (G) Representative immunoblots for stable BUMPT cells expressing Scr-shRNA or *Nat10*-shRNA and transfected with an empty vector or a Pfkm overexpression plasmid, then exposed to HG treatment for 48 h. (H) Immunoblot bands were assessed by densitometric analysis. (I) Relative pyruvate levels in BUMPT cells following transfection with Scr/*Nat10*-shRNA and vector/*Pfkm*-plasmid, then subjected to 48 h HG treatment. (J) Relative lactate levels in BUMPT cells following transfection with Scr/*Nat10*-shRNA and vector/*Pfkm*-plasmid, then subjected to 48 h HG treatment. # P < 0.05 versus Scr-shRNA/NG group. * P < 0.05, versus Scr-shRNA /HG group. ^ P < 0.05, versus *Nat10*-shRNA /HG group. Data are presented as mean ± SD (n = 6).

**Figure 6:**
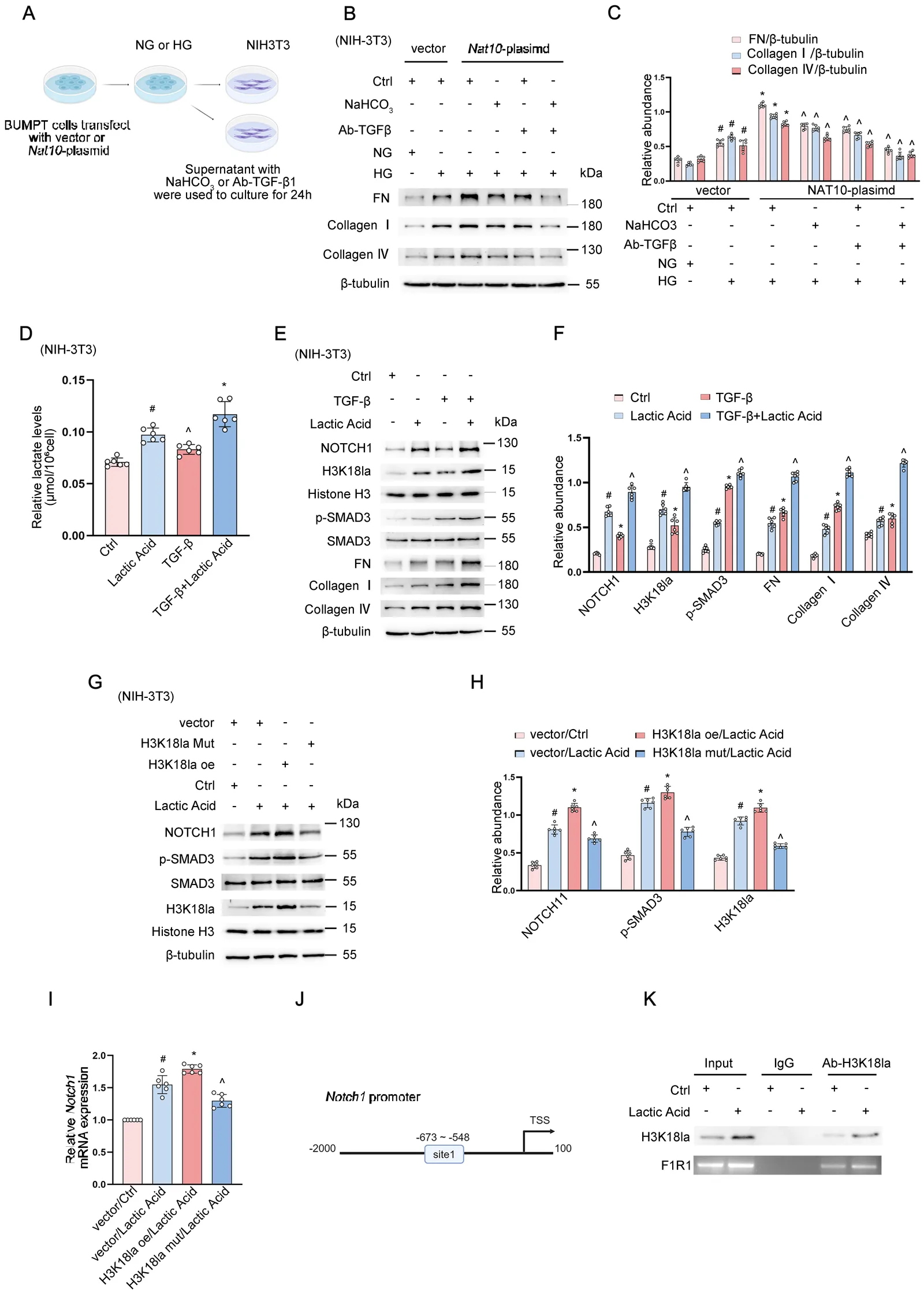
** NAT10 promotes TGF-β1 and lactate production in BUMPT cells, thereby activating mouse kidney fibroblasts through the NOTCH1/SMAD3 pathway.** (A) Schematic diagram conditioned-medium experiment in which NIH/3T3 fibroblasts were cultured with supernatants from HG-treated BUMPT cells. (B, C) BUMPT cells underwent transfection with vector/*Nat10*-plasmid, followed by HG treatment for 48 h. Conditioned medium, with or without NaHCO3 or a TGF-β-neutralizing antibody, was then applied to NIH/3T3 cells. (B) Representative immunoblot analysis for fibrotic markers FN, Collagen I and Collagen IV in NIH/3T3 cell lysates. (C) Immunoblot bands were assessed by densitometric analysis. # P < 0.05 versus Vector/NG group. * P < 0.05, versus Vector/HG group. ^ P < 0.05, versus *Nat10*-plasmid /HG group. (D) Relative lactate levels in NIH/3T3 cells after treatment with TGF-β1 or lactate for 24 h. # P < 0.05 versus Ctrl group. * P < 0.05, versus Ctrl group. ^ P < 0.05, versus Lactic Acid group and TGF-β group. (E) Representative immunoblot analysis for Histone NOTCH1, p-SMAD3, SMAD3, H3K18la, Histone H3, and fibrotic markers FN, Collagen I and Collagen IV in NIH/3T3 cells under lactate or TGF-β treatment. (F)Immunoblot bands were assessed by densitometric analysis. # P < 0.05 versus Ctrl group. * P < 0.05, versus Ctrl group. ^ P < 0.05, versus Lactic Acid group and TGF-β group. (G) H3K18la oe: overexpression of wild-type histone H3 to enhance H3K18 lactylation; H3K18la Mut: H3K18R mutant that abolishes lactylation at this site. Representative immunoblot analysis of NOTCH1, p-SMAD3, SMAD3, H3K18la, and Histone H3 in NIH/3T3 cells following lactate treatment after transfection with vector/H3K18la-plasmid/H3K18la mutant. (H) Immunoblot bands were assessed by densitometric analysis. (I) Relative mRNA expression of *Notch1* was quantified by RT-qPCR. # P < 0.05 versus vector/Ctrl group. * P < 0.05, versus vector/Lactic Acid group. ^ P < 0.05, versus vector/Lactic Acid group. Data are presented as mean ± SD (n = 6). (J) Schematic representation of the Notch1 promoter showing the region from -673 to -548 bp relative to the transcription start site (TSS). (K) ChIP assay showing the enrichment of H3K18la at the *Notch1* promoter.

**Figure 7:**
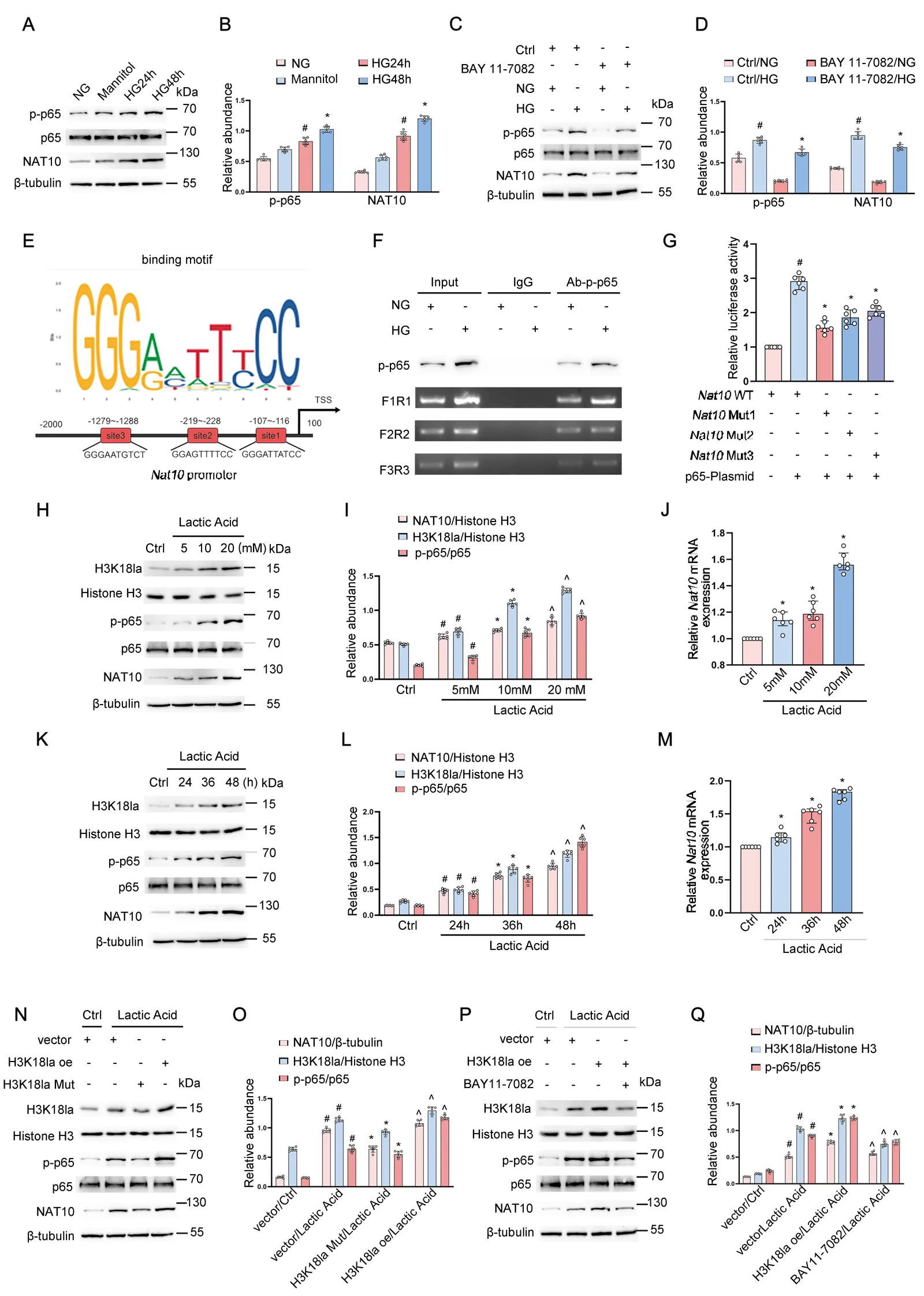
** NAT10 upregulation under HG conditions is mediated by NF-κB and enhanced by lactate.** (A) Representative immunoblot analysis for p-p65 across different time points during HG treatment. (B) Immunoblot bands were quantified through densitometric analysis. # P < 0.05 versus the NG group. * P < 0.05, versus the NG group. (C) Representative immunoblot analysis for p-p65 and NAT10 in BUMPT cells cultured under NG or HG conditions with or without BAY11-7082 treatment. (D) Immunoblot bands were quantified through densitometric analysis. # P < 0.05 versus Ctrl/NG group. * P < 0.05, versus Ctrl/HG group. (E) Schematic illustration of the predicted NF-κB binding site within the *Nat10* promoter. (F) ChIP-PCR analysis examining the p-p65 binding to the *Nat10* promoter. (G) Luciferase reporter assay after co-transfection with the indicated constructs. # P < 0.05 versus *Nat10* WT group * P < 0.05 versus *Nat10* WT/p-65 plasmid group. (H) Representative immunoblot analysis for H3K18la, Histone H3, p-p65, p65, and NAT10 in BUMPT cells subjected to lactate treatment (5, 10, and 20 mM) for 24 h. (I) Immunoblot bands were quantified through densitometric analysis. # P < 0.05 versus Ctrl group. * P < 0.05 versus Ctrl group. ^ P < 0.05 versus Ctrl group. (J) qPCR analysis of *Nat10* mRNA levels after lactate treatment (5, 10, and 20 mM) for 24 h. * P < 0.05 versus Ctrl group. (K)Representative immunoblot analysis for H3K18la, Histone H3, p-p65, p65, and NAT10 in BUMPT cells subjected to lactate treatment (20 mM) for 24, 36, 48 h. (L) Immunoblot bands were quantified through densitometric analysis. # P < 0.05 versus Ctrl group. * P < 0.05, versus Ctrl group. ^ P < 0.05, versus Ctrl group. (M) qPCR analysis of *Nat10* mRNA levels after lactate treatment (20 mM) for 24, 36, 48 h. * P < 0.05 versus Ctrl group. (N) Representative immunoblot analysis for H3K18la, Histone H3, p-p65, p65, and NAT10 in BUMPT cells subjected to lactate treatment after transfection with vector/H3K18la-oe/H3K18la mutant. (O) Immunoblot bands were quantified through densitometric analysis. # P < 0.05 versus vector/Ctrl group. * P < 0.05, versus vector/Lactic Acid group. ^ P < 0.05, versus vector/Lactic Acid group. (P) Representative immunoblot analysis for H3K18la, Histone H3, p-p65, p65, and NAT10 in BUMPT cells subjected to lactate treatment with or without BAY11-7082 after transfection with vector/H3K18la-oe. (Q) Immunoblot bands were quantified through densitometric analysis. # P < 0.05 versus vector/Ctrl group. * P < 0.05, versus vector/Lactic Acid group. ^ P < 0.05, versus vector/Lactic Acid group. Data are presented as mean ± SD (n = 6).

**Figure 8:**
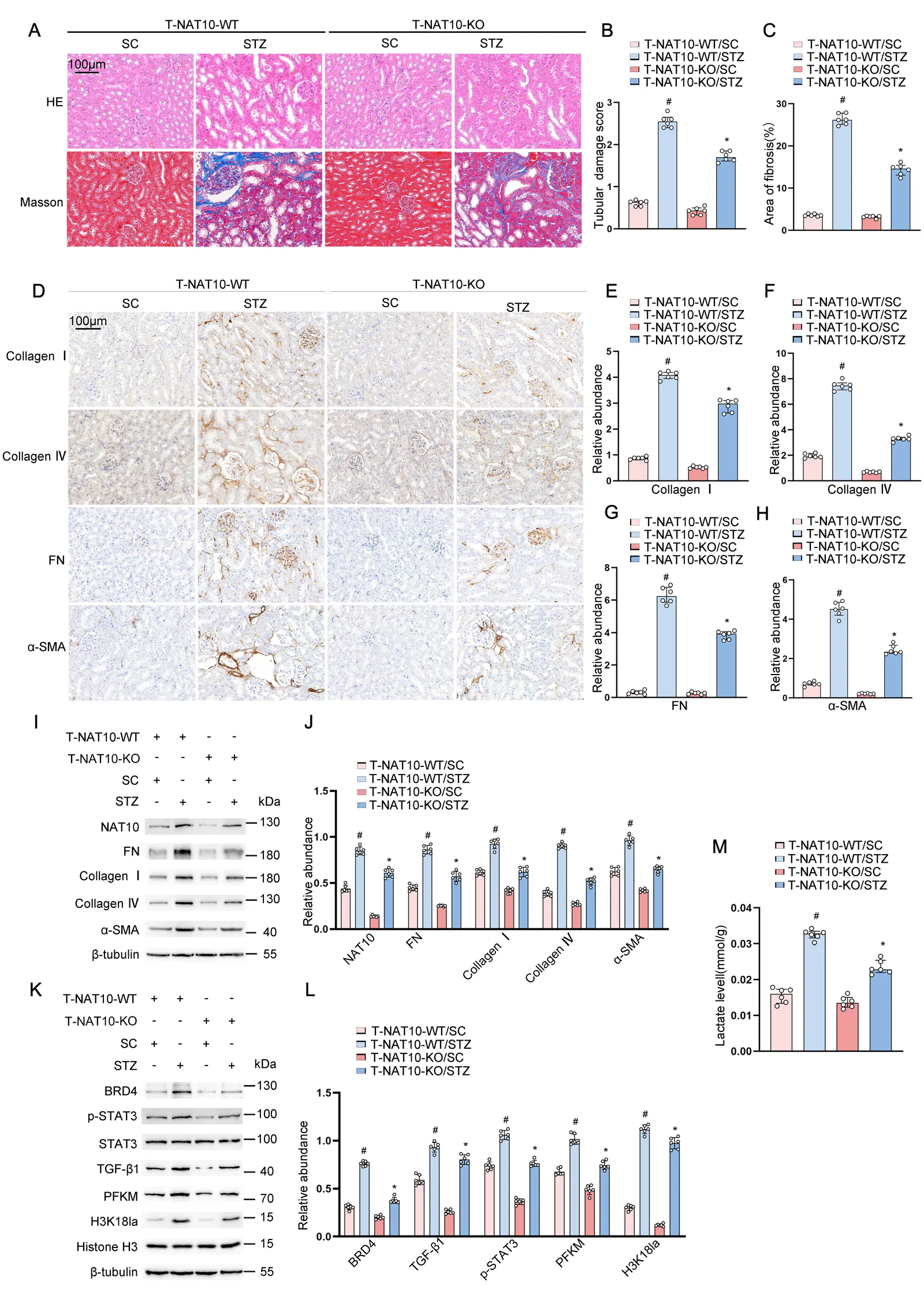
** Tubule-specific deletion of *Nat10* attenuates renal fibrosis in STZ-induced diabetic mice by suppressing BRD4/STAT3/TGF-β1 and PFKM/lactate/H3K18la axes.** Littermate T-NAT10-WT and T-NAT10-KO mice of identical age and weight were assigned to groups receiving either SC or STZ intraperitoneal injection following previously described protocols. (A) H&E staining and Masson staining for fibrosis. Scale bars, 100 μm. (B) Tubular damage score. (C) Fibrosis area. (D) Immunohistochemistry staining for fibrotic markers FN, Collagen I, Collagen IV and α-SMA. Scale bars, 100 μm.(E-H) Analysis of immunohistochemistry staining. (I) Immunoblot analysis for NAT10 and fibrotic markers FN, Collagen I, Collagen IV and α-SMA in mice kidneys from diverse groups. (J) Immunoblot bands were quantified through densitometric analysis. (K) Immunoblot analysis for BRD4, p-STAT3, STAT3, TGF-β1, PFKM, H3K18la, and Histone H3 in mice kidneys from diverse groups. (L) Immunoblot bands were quantified through densitometric analysis. (M) Relative lactate levels within the kidney. # P < 0.05 versus T-NAT10-WT/SC. * P < 0.05 versus T-NAT10-WT/STZ. Data are presented as mean ± SD (n = 6).

**Figure 9:**
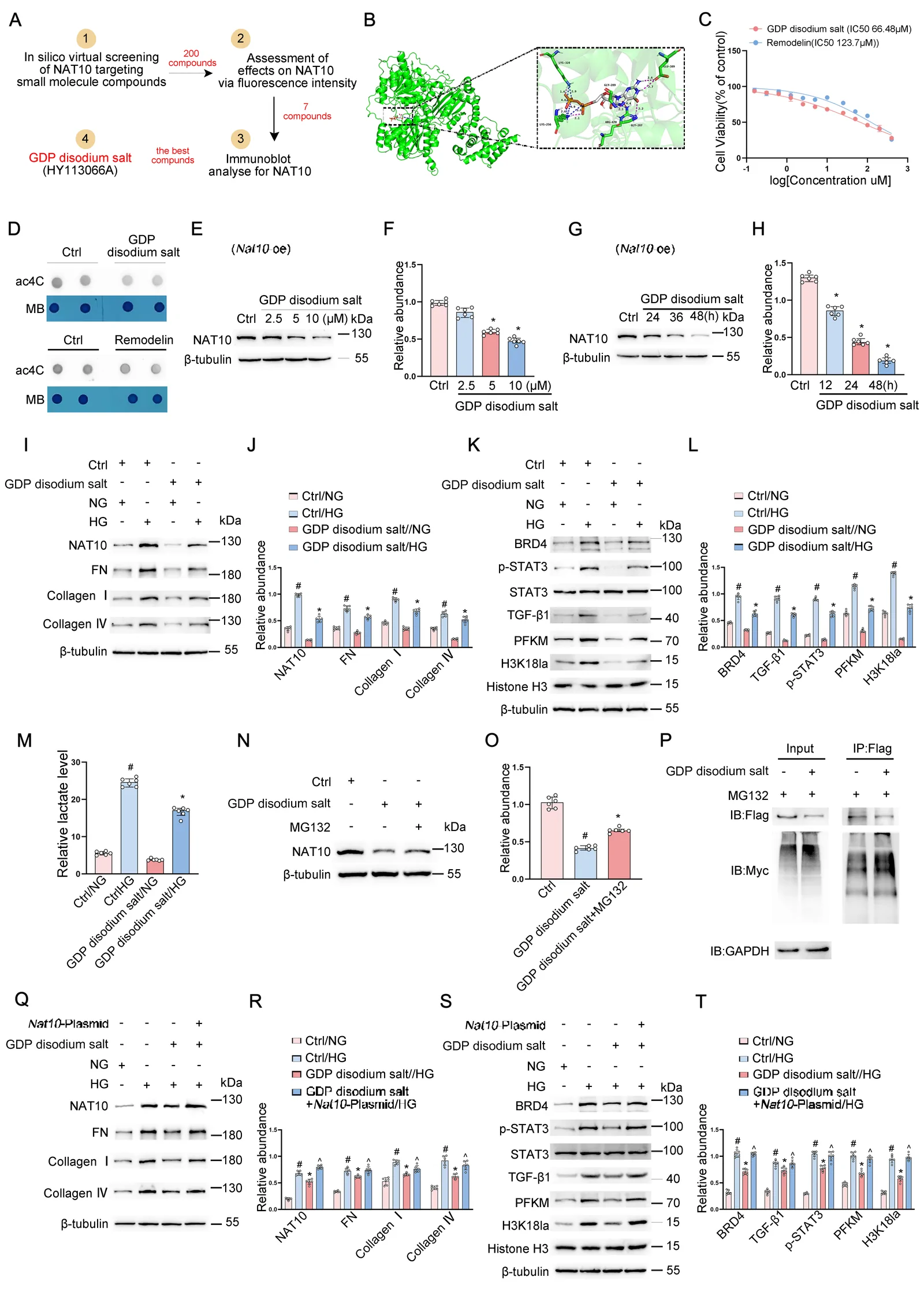
** Pharmacological inhibition of NAT10 attenuates high-glucose-induced profibrotic responses in BUMPT cells.** (A) Flowchart for small-molecule compound screening. (B) Molecular docking between NAT10 and GDP disodium salt. (C) CCK-8 assays and IC50 values for GDP disodium salt and remodelin in BUMPT cells after 48 h treatment. (D) Dot blotting showing RNA ac4C reduction in BUMPT cells with GDP disodium salt or remodelin treatment. Methylene blue staining was employed as an RNA loading control. (E, G) Immunoblot showing NAT10 protein levels in stable *Nat10*-oe BUMPT cell line with GDP disodium salt treatment at 2.5 μM, 5 μM, 10 μM, and 24 h, 36 h, 48 h. (F, H) Immunoblot bands were quantified through densitometric analysis. * P < 0.05 versus Ctrl group. (I) Immunoblot analysis for NAT10 and fibrotic markers FN, Collagen I, Collagen IV in BUMPT cells with GDP disodium salt and HG incubation treatment for 48 h. (J) Immunoblot bands were quantified through densitometric analysis. (K) Immunoblot analysis for BRD4, p-STAT3, STAT3, TGF-β1, PFKM, H3K18la, and Histone H3 in BUMPT cells with GDP disodium salt and HG incubation treatment for 48 h. (L) Immunoblot bands were quantified through densitometric analysis. # P < 0.05 versus the Ctrl/NG group. * P < 0.05 versus the Ctrl/HG group. (M) Relative medium lactate level in BUMPT cells. # P < 0.05 versus the Ctrl/NG group. * P < 0.05 versus the Ctrl/HG group. (N) Immunoblot analysis for NAT10 protein levels in BUMPT cells with or without MG132 treatment. (O) Immunoblot bands were quantified through densitometric analysis. # P < 0.05 versus Ctrl group. * P < 0.05 versus GDP disodium salt group. (P) Cells were co-transfected with Flag-NAT10 and Myc-ubiquitin. Cell lysates were immunoprecipitated with anti-Flag antibody and analyzed by immunoblotting with the indicated antibodies. (Q,S) Immunoblot analysis for NAT10, FN, collagen I, collagen IV, BRD4, TGF-β1, STAT3, p-STAT3, PFKM, and H3K18la in cells cultured under NG or HG conditions and transiently transfected with the *Nat10* overexpression plasmid, followed by treatment with GDP disodium salt as indicated. β-tubulin or histone H3 served as the loading control. (R, T) Immunoblot bands were quantified through densitometric analysis. # P < 0.05 versus the Ctrl/NG group. * P < 0.05 versus the Ctrl/HG group. ^ P < 0.05 versus the GDP disodium salt/HG group. Data are presented as mean ± SD (n = 6).

**Figure 10:**
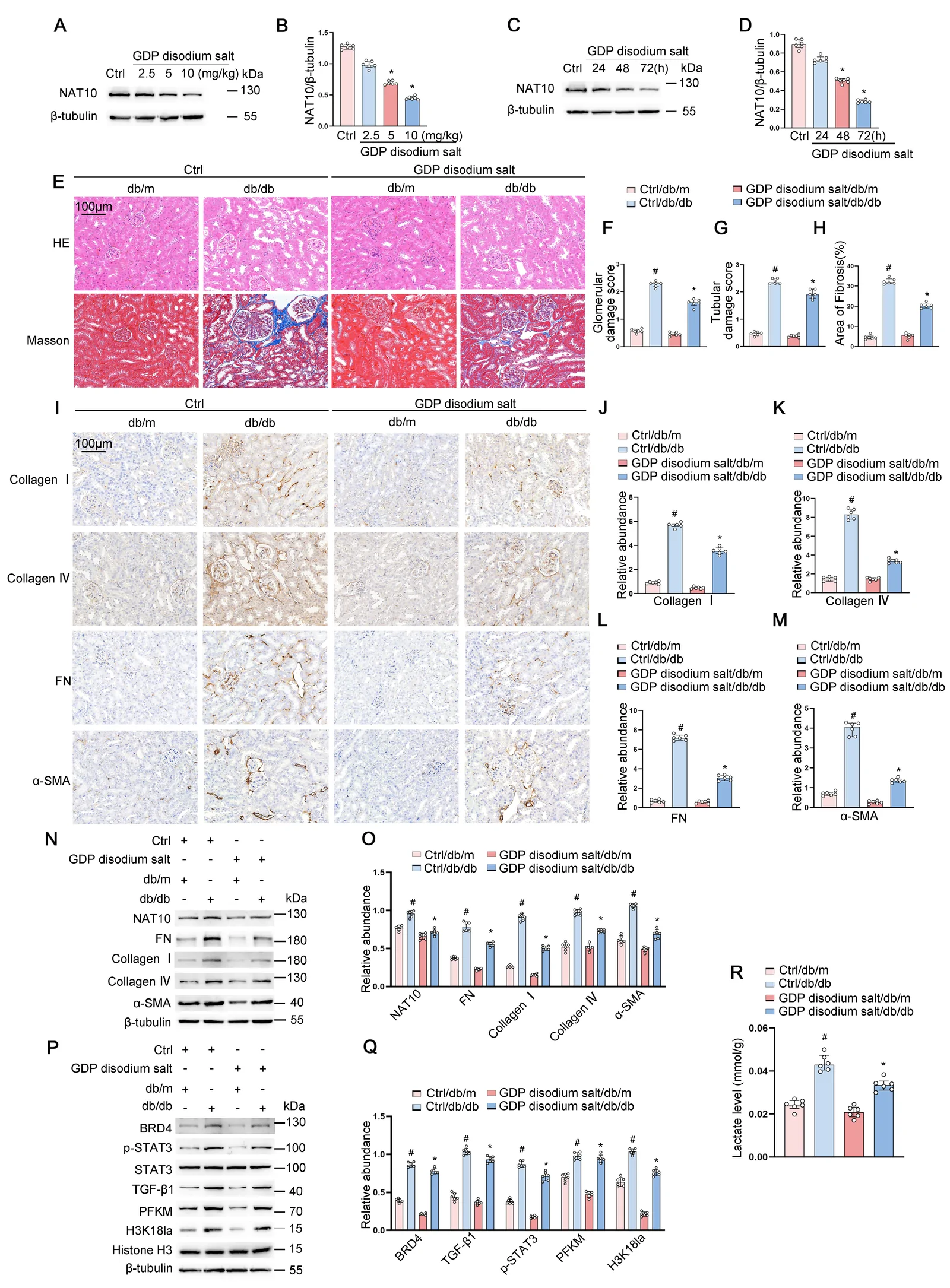
** GDP disodium salt attenuates diabetes-induced renal fibrosis in *db/db* mice by inhibiting NAT10.** Eight-week-old *db/db* and *db/m* mice were intraperitoneally injected with GDP disodium salt (10 mg/kg) or PBS once every 3 days until 18 weeks of age. (A, C) Immunoblot analysis for NAT10 in C57BL/6 mice with GDP disodium salt treatment at 2.5 mg/kg, 5 mg/kg, 10 mg/kg, and 24 h, 48 h, 72 h. (B, D) Immunoblot bands were quantified through densitometric analysis. * P < 0.05 versus ctrl group. (E) H&E staining and Masson staining for fibrosis. Scale bars, 100 μm. (F) Glomerular damage score. (G) Tubular damage score. (H) Fibrosis area. (I) IHC staining for fibrotic markers FN, Collagen I, Collagen IV, and α-SMA. Scale bars, 100 μm. (J-M) Analysis of IHC staining. (N) Immunoblot analysis for NAT10 and fibrotic markers FN, Collagen I, and Collagen IV in* db/db* mice with GDP disodium salt treatment for 10 weeks. (O) Immunoblot bands were quantified through densitometric analysis. (P) Immunoblot analysis for BRD4, p-STAT3, STAT3, TGF-β1, PFKM, H3K18la, and Histone H3 in *db/db* mice with GDP disodium salt treatment for 10 weeks. (Q) Immunoblot bands were quantified through densitometric analysis. (R) Renal lactate levels. # P < 0.05 versus ctrl/*db/m*. * P < 0.05 versus ctrl/*db/db*. Data are presented as mean ± SD (n = 6).

## Data Availability

The ac4C-RIP-seq data generated in this study have been deposited in the GEO database (GSE337861) and will be publicly available upon publication.
