# Supplementary material for: Tubular NAT10 Promotes the Secretion of TGF-β and Lactate to Drive Fibroblast Activation in Diabetic Kidney Disease
Source: Int J Biol Sci. 2026 Sep 2;22(14):7737–59. doi: 10.7150/ijbs.140281 (PMC13617997; doi:10.7150/ijbs.140281)
Supplement: Supplementary file 1 [file ijbsv22p7737s1.pdf]

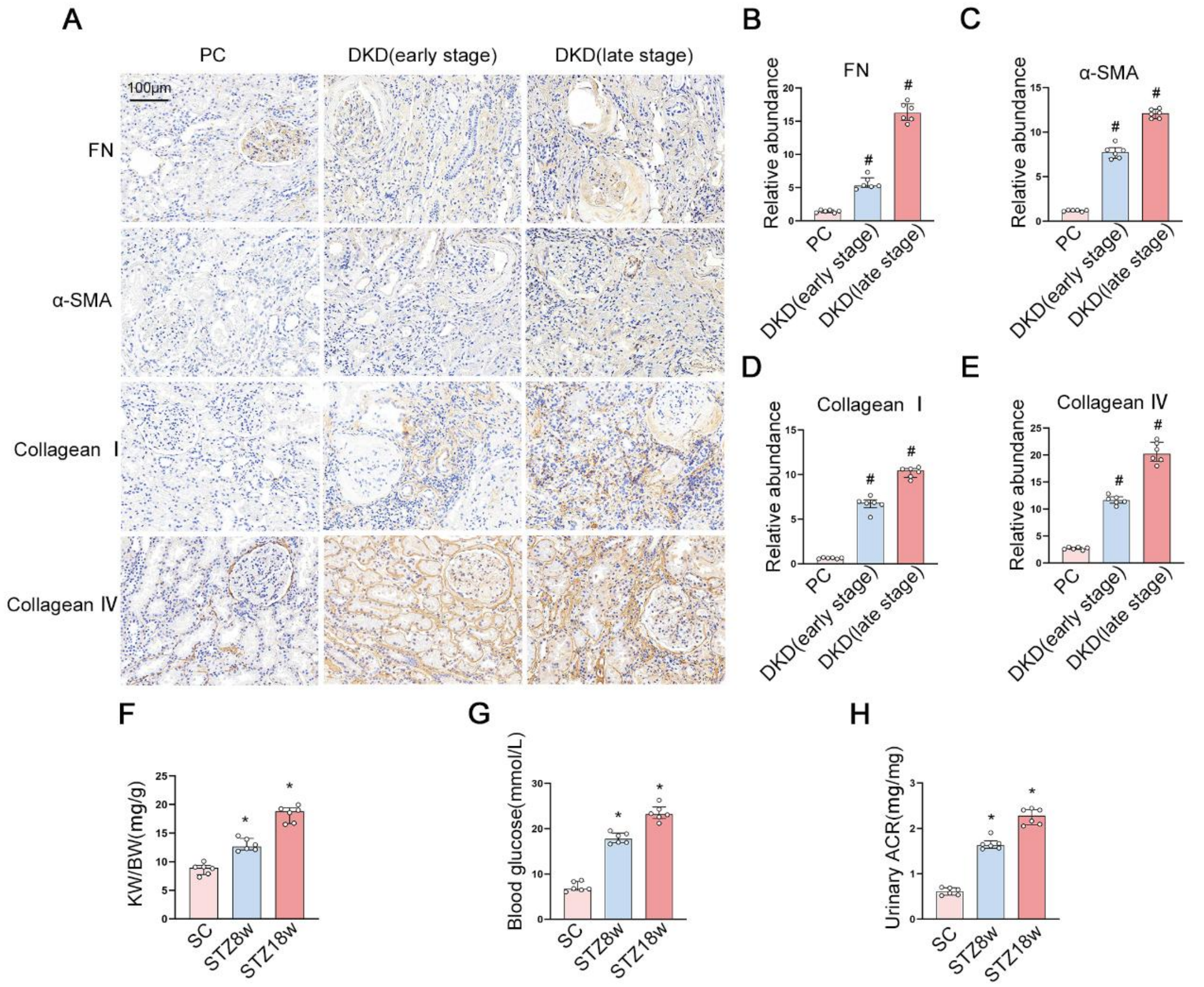

Supplementary Figure S1: Fibrotic markers in kidney biopsies of human DKD patients and characters of STZ-induced diabetes in mice.

(A)Immunohistochemistry staining of fibrotic proteins. Original magnification x400. Scale Bar:100µm. (B-E) Quantify immunohistochemistry staining. (F) kidney to total body weight ratio (KW/BW) for 18 weeks, 8 weeks STZ-induced diabetes mice and control (SC) mice for analysis. C57BL/6 mice were intraperitoneally injected with 50 mg/kg body weight STZ for 5 consecutive days to induce diabetes or injected with sodium citrate (SC) as a control. Samples were collected at 18 weeks or 8 weeks after diabetes induction for analysis. (G) Fasting blood glucose. (H) Urinary albumin to creatinine ratio (ACR).

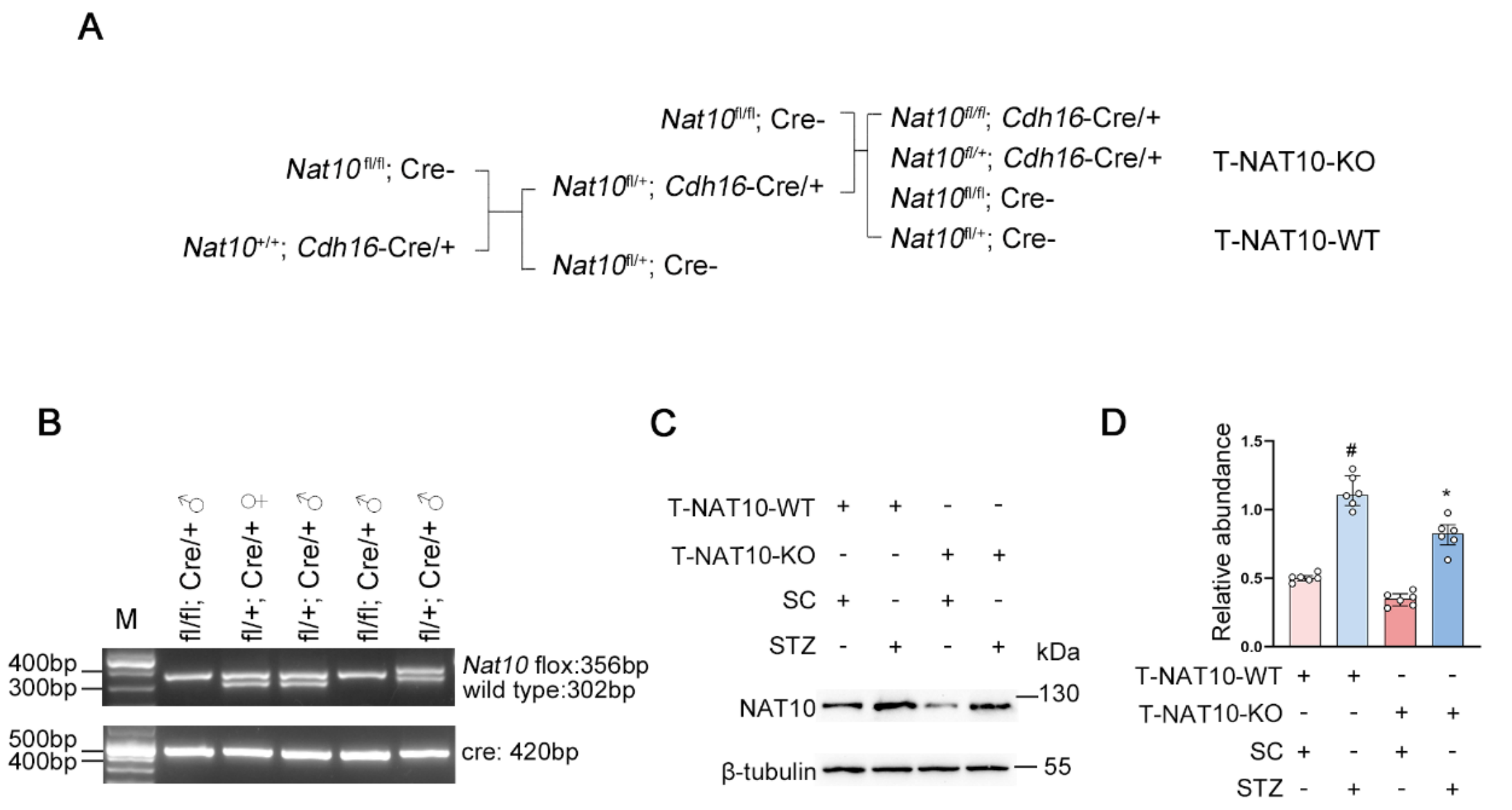

Supplementary Figure S2: Creation and characterization of the T-NAT10-KO mouse model. (A)Breeding protocol for the creation of T-NAT10-KO mice. (B) PCR detection of wild-type and floxed alleles of *Nat10* and *Cdh16*-Cre allele. Lane M: 100 bp DNA molecular weight ladder (unit: bp). (C) Immunoblot analysis of NAT10 and β-tubulin. (D) Densitometry analysis of immunoblot bands. Littermate mice of T-NAT10-WT and T-NAT10-KO were intraperitoneally injected with STZ at a dose of 50 mg/kg body weight for 5 consecutive days or sodium citrate (SC) as a control. Mice with fasting blood glucose levels > 200 mg/dL on two consecutive measurements were defined as diabetic. Samples were collected 18 weeks after diabetes induction for analysis. Data are expressed as mean ±SD (n = 6). #P<0.05, versus SC-treated T-NAT10-WT group; * P<0.05, versus STZ-treated T-NAT10-WT group.

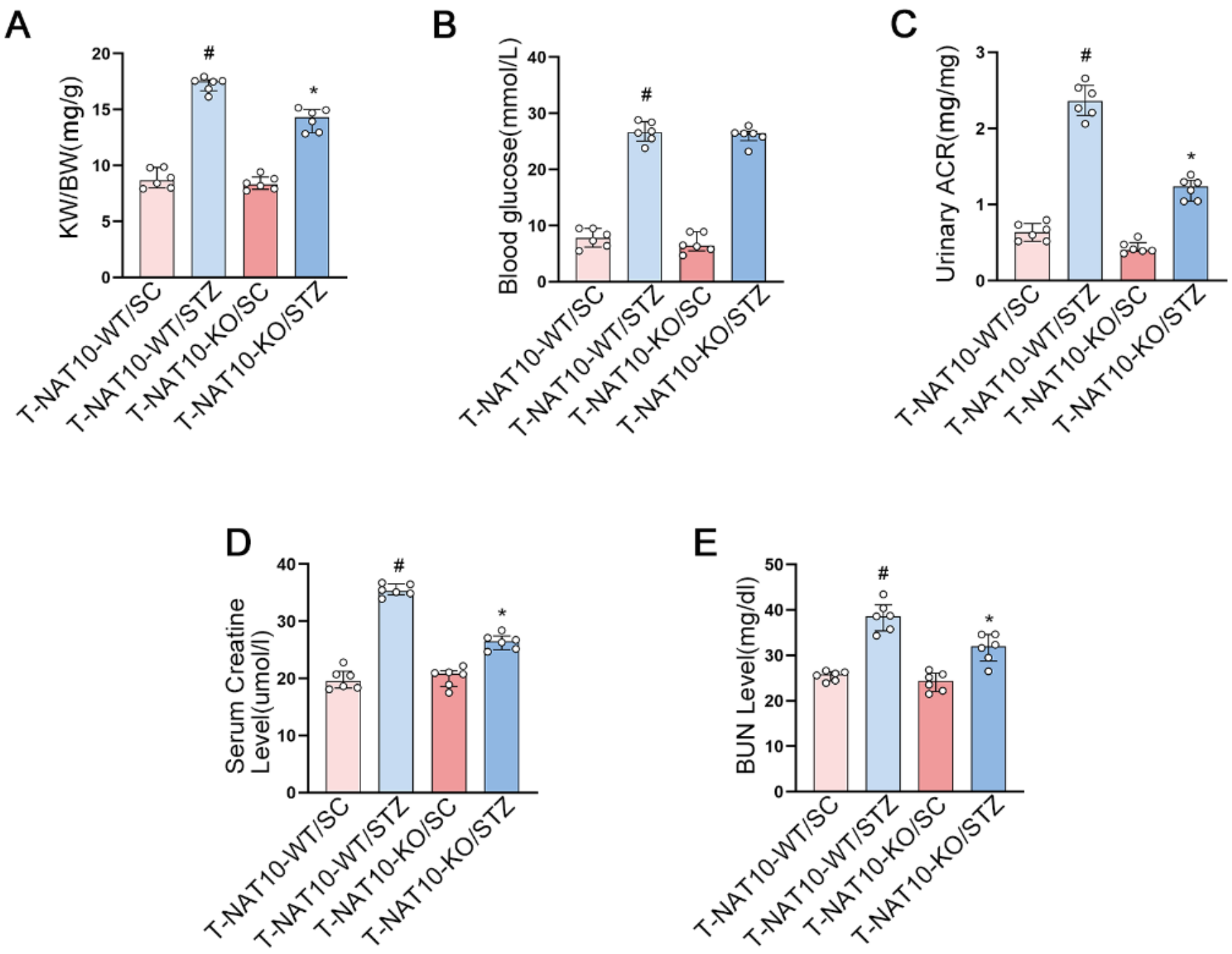

Supplementary Figure S3: NAT10-knockout from kidney proximal tubules attenuates renal functions in STZ-induced diabetic mice.

Littermate mice of T-NAT10-WT and T-NAT10-KO were intraperitoneally injected with 50 mg/kg body weight STZ or sodium citrate (SC) as a control for 5 consecutive days. The fasting blood glucose levels of more than 200 mg/dL for two consecutive measurements were regarded as diabetic. Samples were collected 18 weeks after diabetes induction for analysis. (A) kidney to total body weight ratio (KW/BW). (B) Fasting blood glucose. (C) Urinary albumin to creatinine ratio (ACR). (D) Serum Creatine Level. (F) Blood Urea Nitrogen (BUN) Level. # $P<0.05$, versus SC-treated T-NAT10-WT group * $P<0.05$, versus STZ-treated T-NAT10-WT group. Data are expressed as mean ± SD (n = 6).

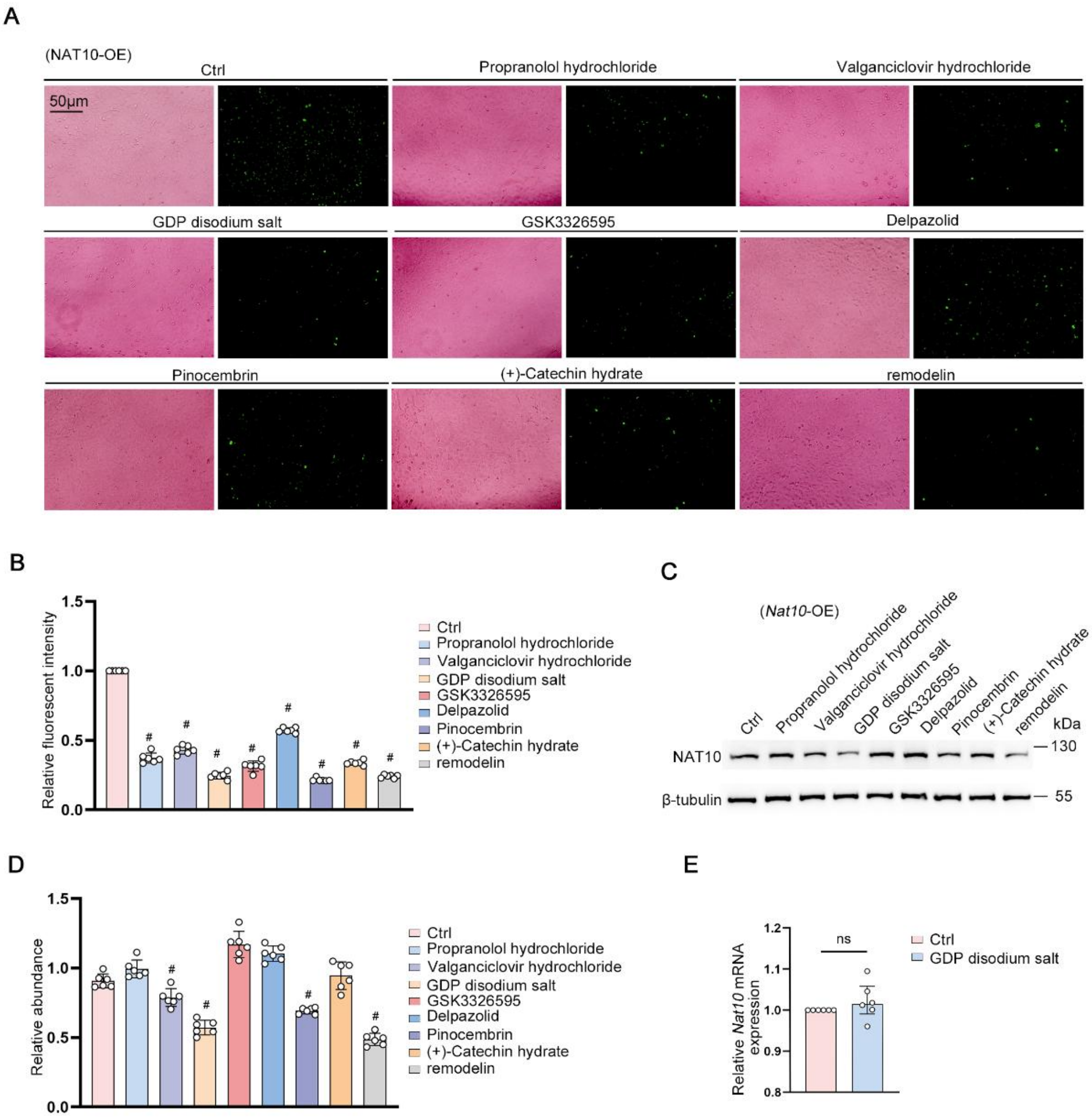

Supplementary Figure S4: Assessment of the effects of small molecule compounds targeting NAT10 in BUMPT cells.

(A) Fluorescence intensity of NAT10-overexpressing BUMPT cells treated with small molecule compounds for 24 h. (B)Relative fluorescence intensity of different small molecule compounds groups and DMSO (control) group. (C) Immunoblot analysis of NAT10 in stable NAT10-overexpressing BUMPT cell lines treated with the indicated compounds for 24 h. (D)Densitometry analysis of immunoblot bands. #P<0.05, versus control group. Scale bars, 50 µm. (E) qPCR analysis of *Nat10* mRNA levels after PBS or GDP disodium salt treatment for 10µM, 48h. Data are expressed as mean ±SD (n = 6).

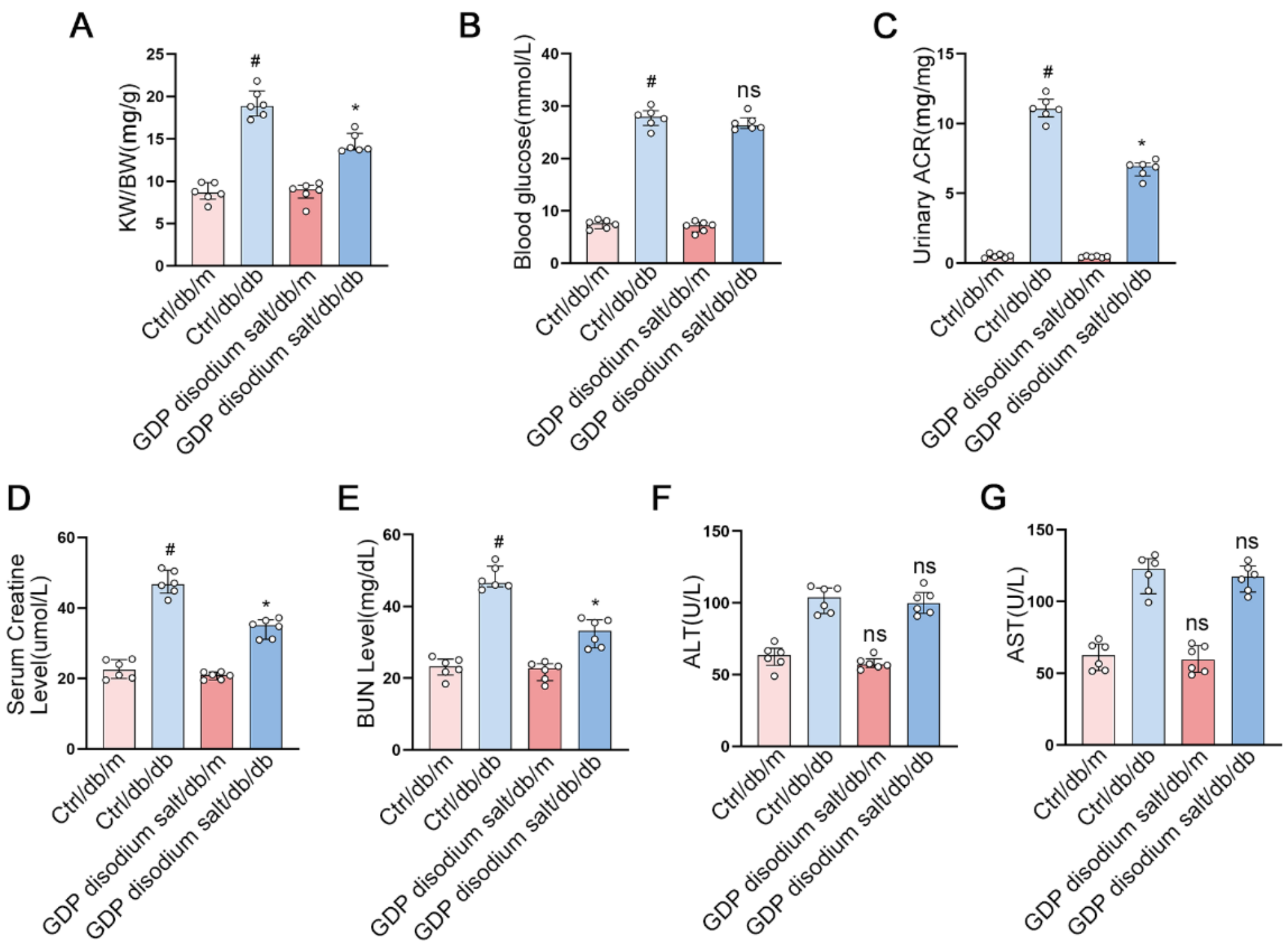

Supplementary Figure S5: inhibitor GDP disodium salt alleviates renal dysfunction in *db/db* diabetic mice.

8-week-old *db/db* mice and *db/m* mice were intraperitoneally injected with GDP disodium salt at 10 mg/kg or PBS as control once every 3 days until 18 weeks of age. The fasting blood glucose levels of more than 200 mg/dL for two consecutive measurements were regarded as diabetic. Samples were collected at 18 weeks of age for analysis. (A) kidney to total body weight ratio (KW/BW). (B) Fasting blood glucose. (C) Urinary albumin to creatinine ratio (ACR). (D) Serum Creatine Level. (E) Blood Urea Nitrogen (BUN) Level. # P<0.05, versus Ctrl/*db/m*. * P<0.05, versus Ctrl/*db/db* group. ns, versus Ctrl/*db/db* group. (F–G) Serum alanine aminotransferase (ALT) and aspartate aminotransferase (AST) levels in *db/m* and *db/db* mice treated with PBS or GDP disodium salt. ns, GDP disodium salt-treated *db/m* versus Ctrl/*db/m*, or GDP disodium salt-treated *db/db* versus Ctrl/*db/db*. Data are expressed as mean ± SD (n = 6).

Supplementary Table 1. Baseline clinical characteristics of pathological control group and patients with early-stage and late-stage DKD.

Variable	PC (n=9)	DKD (early stage) (n=13)	DKD (late stage) (n=12)
Age, years, mean ± SD	56.78±8.74	52.77±11.78	62.00±6.41
Sex, male/female	6：3	7：6	8：4
Serum glucose, mmol/L, mean ± SD	5.92±1.24	8.04±1.70$^{\#}$	10.53±0.99*
eGFR, mL/min/1.73 m², mean ± SD	101±6.05	76.47±13.87$^{\#}$	12.15±6.31*
UACR, mg/g, mean ± SD	NA	152.8±64.73	3640±1458†

Data are presented as mean ± SD or n. urinary albumin-to-creatinine ratio (UACR) data were not available for the pathological control (PC) group and are therefore shown as NA. UACR is included only

to describe albuminuria severity in the DKD subgroups. #P < 0.05 vs. PC group; *P < 0.05 vs. early-stage DKD group for variables with available data in all three groups; † P < 0.05 vs. early-stage DKD group for UACR.

Supplementary Table 2. Full list of *Nat10*-regulated genes under high-glucose conditions

This supplementary file provides the complete list of *Nat10*-regulated genes identified under high-glucose conditions. Genes were selected based on the following criteria: upregulated in the Scr-shRNA/HG group compared with the Scr-shRNA/NG group, and downregulated in the *Nat10* shRNA/HG group compared with the Scr-shRNA/HG group. For each gene, fold change and adjusted p value are shown for both comparisons. *Brd4* and *Pfkm*, the key genes highlighted in this study, are specifically annotated.

GeneName	Fold change (up)	Adjusted P value	Fold change (down)	Adjusted P value	Annotation
Brd4	**691.20**	**2.66289E-11**	**231.20**	**1.61773E-10**	**Key gene**
Pfkm	**170.00**	**1.09086E-06**	**200.60**	**1.10899E-07**	**Key gene**
Supt6	2842.50	1.09109E-07	2.83	1.67869E-05	
Mepce	2485.50	1.54384E-09	2924.90	4.698E-09	
Dhx9	2393.90	5.79619E-10	2816.00	1.11734E-10	
Prrc2c	2359.00	9.51408E-09	5.02	1.09873E-07	
Gm12184	2228.80	3.51076E-07	234.48	5.70809E-07	
Gm6169	1968.50	1.22138E-10	2313.60	6.68587E-11	
Trim3	1954.40	1.60025E-10	284.20	1.01477E-07	
Arhgdia	1761.50	3.10047E-09	2071.00	1.36628E-10	
Adnp	1654.90	1.82726E-07	2269.80	1.30745E-07	
Mtch1	1407.90	3.93458E-08	642.90	3.152E-09	
Mrpl28	1388.40	9.39191E-10	1633.00	5.64199E-11	
Arf1	1384.60	9.10128E-10	2.31	2.9905E-05	
Nop58	1323.70	6.40948E-08	1558.40	9.43089E-08	
Tmem171	1316.30	1.80877E-10	2.30	3.15254E-05	
Map1b	1305.40	4.22708E-08	352.70	4.50606E-12	
Mcm5	1223.50	9.64089E-08	688.30	1.94278E-07	
Dhx29	1123.90	2.49298E-10	8.87	3.3182E-11	
Asap3	1115.40	1.75191E-10	3.13	8.40266E-05	
Ptprf	1065.70	1.88342E-08	2.24	3.75389E-05	
Sdc1	1064.00	1.37363E-10	2.93	1.26789E-05	
Dcakd	1025.40	1.23916E-10	1206.00	2.55044E-11	
Tuba1a	1017.50	5.60746E-08	1197.90	7.77597E-08	
Txndc12	1006.70	1.58944E-09	1184.60	2.85189E-09	
Safb2	989.50	1.48217E-07	1161.60	1.91808E-07	
Foxj3	964.20	4.18597E-11	185.75	2.36459E-11	
Ep300	950.30	4.82454E-08	28.06	9.07104E-08	
Nos2	927.40	6.25299E-11	2.28	6.33745E-05	
Gpank1	922.50	3.46663E-11	1084.10	1.946E-11	
Gmeb2	897.30	8.57346E-09	317.30	3.06301E-08	

Eri3	893.80	5.59106E-11	328.70	4.91163E-08
Itgb4	887.80	1.36254E-07	216.88	8.42787E-08
C1qtnf1	870.60	4.32066E-11	447.90	4.22246E-11
1110002E22Rik	861.20	1.73506E-07	962.20	9.40779E-08
Piezo1	845.20	4.76616E-11	78.02	2.73501E-07
Sae1	839.00	3.24505E-11	986.00	1.67757E-11
Senp3	794.40	1.84646E-07	384.70	1.21771E-07
Ap2b1	791.30	1.73704E-07	928.50	2.62081E-07
Cwc22	786.70	8.60627E-08	120.10	1.70897E-05
Mypop	775.30	1.77975E-07	909.60	2.7773E-07
Pnpla6	771.70	1.50116E-07	905.70	2.11477E-07
Cdh6	766.40	1.33831E-07	8.36	3.69055E-05
Card10	762.00	1.3718E-07	2.06	4.35234E-05
Phactr4	757.10	2.40075E-11	890.00	4.8109E-12
Xpa	749.50	2.33559E-11	753.20	2.03996E-07
Rwdd4a	739.50	4.11841E-11	512.20	7.69569E-08
Maco1	736.40	3.39561E-10	580.70	1.5017E-10
Pdpk1	722.70	2.75102E-08	75.79	2.26728E-08
Mdn1	720.60	2.68508E-11	711.00	2.06316E-07
Tusc1	710.70	7.00984E-11	835.00	1.95849E-11
Baz1a	705.00	2.78371E-11	4.56	1.25073E-06
Galnt2	700.50	7.84573E-08	762.80	9.28643E-08
Syvn1	682.70	2.53243E-11	802.50	2.00364E-12
Pfkp	682.30	1.21103E-09	802.90	1.78543E-09
Kif1b	681.50	1.23905E-07	886.70	9.20312E-08
Stam	676.90	1.09957E-07	70.95	1.96824E-07
Kif1c	663.70	1.21083E-07	2.60	2.01901E-05
Hexb	662.90	1.04423E-07	780.70	1.55262E-07
Med26	649.70	6.96994E-08	13.23	1.81556E-06
Xbp1	643.9	6.20753E-11	756.50	1.65641E-11
Mdm1	625.00	2.33559E-11	144.60	3.07762E-06
Adap1	621.90	1.19971E-07	419.40	1.03064E-07
Xntrpc	621.90	2.17706E-11	730.80	3.63187E-12
Hivep2	620.50	2.33559E-11	729.30	2.00364E-12
Sec22b	617.70	4.18421E-11	726.70	9.64164E-11
Rock1	612.30	2.4272E-11	8.20	3.05334E-06
Il3ra	611.40	1.24983E-07	244.10	4.04802E-08
Gns	592.70	7.23236E-08	694.10	9.88025E-08
Cep152	580.40	1.40973E-07	106.80	9.35742E-05
Knl1	577.30	2.33559E-11	678.60	2.00364E-12
Timeless	576.00	3.21457E-10	551.10	9.10572E-08
Clip2	573.60	2.33559E-11	2.30	1.23958E-04
Numa1	573.30	1.67015E-10	673.50	3.49252E-11
Ubl7	566.60	8.72707E-08	8.63	7.75987E-06

Mtss2	565.70	1.64056E-09	33.39	9.79243E-10
Zfp358	563.50	1.93394E-11	9.27	8.01968E-12
Trmt44	550.40	1.67621E-08	646.50	6.36278E-09
Usp22	546.40	3.00748E-09	384.70	1.02039E-07
Cenpe	536.60	7.70149E-08	7.15	4.0608E-08
Csrp1	534.80	8.40968E-08	25.52	4.99472E-07
Pias4	527.00	4.62107E-11	619.20	1.3055E-11
Neo1	526.90	7.16256E-08	246.70	8.48996E-08
Tmbim1	523.90	1.77683E-09	620.00	1.42948E-10
Mdc1	522.6	1.94702E-11	173.41	3.57125E-07
Erbb2	516.60	1.95861E-11	113.40	3.97608E-06
Cep164	513.90	6.65057E-08	605.10	8.96726E-08
Akr1b8	512.10	1.96223E-11	602.30	3.44545E-12
Huwe1	507.97	5.25874E-08	618.28	6.15345E-10
Ephb6	506.10	3.3495E-10	595.40	5.16311E-10
Tgfbrap1	505.60	4.97993E-08	593.70	2.72111E-08
Abcd4	501.00	3.87953E-08	588.40	2.40017E-08
Anxa4	500.90	1.24333E-07	587.80	1.43241E-07
E2f1	496.10	1.90179E-11	3.17	1.20989E-04
Thoc5	494.00	1.2098E-07	579.50	1.6652E-07
Abcc1	491.90	9.01724E-08	579.30	1.29012E-07
Tap2	491.40	1.90079E-11	30.30	2.82085E-07
Hps4	491.20	8.06821E-09	577.00	2.43227E-09
Yif1b	486.90	1.90079E-11	410.40	1.43194E-07
Cep170b	486.40	1.41827E-07	2.17	9.08259E-05
Gle1	484.90	2.44281E-09	1178.70	2.76932E-11
Spred2	478.20	8.44435E-08	807.00	2.30405E-07
Wdr5	477.2	5.00494E-10	208.4	2.2521E-09
Igsf3	476.90	4.34837E-11	50.03	1.38262E-11
Phc3	475.40	1.90079E-11	558.70	2.00364E-12
Plk1	474.70	8.29001E-08	1560.80	1.02942E-10
Phlpp1	473.80	1.10948E-07	558.10	1.66695E-07
Abca7	462.40	1.815E-08	28.41	2.70319E-07
Mif4gd	457.50	2.50098E-11	538.10	5.05431E-11
Cnot3	454.10	7.22968E-08	87.15	1.76003E-07
Zfp703	452.00	1.90079E-11	11.38	5.94532E-12
Mcu	451.30	1.90179E-11	530.70	2.00364E-12
Borcs8	451.10	1.90079E-11	530.10	2.00364E-12
Wdr54	450.70	2.36454E-08	530.50	1.711E-08
Rgma	449.90	1.00825E-07	527.50	2.14487E-07
Fhod3	448.60	7.70524E-08	25.00	3.1824E-07
Dock1	448.60	7.70524E-08	526.50	7.4838E-08
Itpr1	447.30	1.92256E-11	525.60	2.85196E-12
Grk5	445.00	4.89276E-09	183.90	1.49627E-07

Prpf40b	444.80	1.00698E-07	675.30	1.90685E-08
Asb7	443.10	1.04571E-08	135.60	7.30402E-06
Synpo	439.70	1.95383E-10	38.31	2.72098E-09
Nup214	439.10	1.90079E-11	4.54	6.59958E-11
Mppe1	433.50	2.83514E-08	509.10	1.09599E-08
Kif16b	431.70	1.62217E-09	1327.00	3.05359E-11
Lars	430.20	2.36454E-08	506.30	2.2821E-08
Cc2d1a	429.50	1.21358E-07	504.00	1.07475E-07
Ankrd33b	429.00	1.84743E-08	503.80	8.00257E-09
Foxj2	425.00	1.06129E-07	2.18	8.18496E-06
Nudt12	424.60	1.90079E-11	499.10	2.00364E-12
Bin1	423.70	3.03981E-09	287.90	5.09823E-08
Slc25a48	423.50	8.46796E-08	366.40	2.18343E-08
Uggt1	422.60	1.02295E-08	497.30	1.10465E-08
Ipmk	419.20	7.76168E-10	295.80	6.91534E-08
Kdm3a	415.90	6.99869E-08	489.70	6.85677E-08
Zdhhc24	408.90	7.60951E-08	477.70	1.04517E-07
Ankrd13a	406.80	8.46723E-08	2.95	1.37292E-07
Erich1	404.80	1.90079E-11	475.70	2.00364E-12
Zfp428	404.30	6.31164E-08	83.14	2.06316E-07
Dnajc10	403.90	1.00825E-07	475.90	1.87473E-07
Fancm	398.30	9.62106E-08	467.10	1.5558E-07
Nbeal2	397.80	7.16256E-08	489.10	8.72808E-08
Cep162	397.40	1.90079E-11	5.31	1.51375E-07
Bcl3	396.90	1.2495E-07	3.45	1.24859E-05
Ptprs	394.10	8.83628E-10	148.80	1.06844E-06
Prickle1	393.40	2.61325E-11	462.20	6.51303E-12
Znrf3	378.20	7.35179E-08	442.50	1.0122E-07
Ankrd26	372.90	2.05583E-10	388.20	2.68397E-12
Gdf11	370.70	1.90079E-11	2186.80	6.68587E-11
Spn	368.9	9.80589E-08	2.33	3.61126E-06
Scaper	368.50	1.90079E-11	433.30	2.00364E-12
Wdr7	368.40	2.53939E-11	186.10	1.0191E-07
Smarcc1	366.10	7.07435E-08	432.00	9.61652E-08
Glo1	364.40	8.64699E-08	427.60	7.35804E-08
Gorab	362.40	1.90079E-11	425.90	2.00364E-12
Plpp1	358.70	2.02934E-11	422.00	4.00723E-11
Pde4a	353.50	6.46293E-08	414.30	8.94813E-08
Nle1	350.60	7.35909E-08	412.70	6.59641E-08
Man2a1	347.90	2.24282E-10	323.00	9.86061E-08
Rbm47	347.70	8.7031E-08	408.00	7.389E-08
Setd5	342.34	5.86495E-09	2375.00	4.02128E-10
Lats2	341.70	4.50389E-08	401.10	1.98995E-08
Pdcd7	341.60	6.12184E-08	402.70	8.48516E-08

Eapp	339.30	8.49752E-08	399.80	1.85351E-07
Cmas	333.30	4.04939E-08	391.30	2.6573E-08
Ctdp1	331.20	7.41565E-08	12.46	5.92371E-07
Pla1a	330.60	1.90079E-11	388.80	2.15485E-12
Sft2d2	326.60	2.24821E-11	383.70	2.88515E-12
Ccdc191	325.70	6.45887E-08	120.30	1.42619E-05
Pdlim5	311.70	1.46653E-08	296.70	1.51985E-11
Lancl3	307.70	1.90079E-11	361.70	3.58931E-12
Rnf40	304.80	2.16543E-10	16.38	9.07104E-08
Tfb2m	300.30	1.67166E-09	13.38	1.03888E-08
Gmpr	300.10	1.90079E-11	352.70	4.50606E-12
Fxn	296.60	8.0625E-08	349.30	9.48025E-08
Porcn	294.80	1.90079E-11	346.60	4.69186E-12
Tamalin	294.00	7.48963E-08	11.69	6.11537E-07
Clpx	293.40	1.90079E-11	345.00	4.26941E-12
Osbpl2	291.90	3.20681E-09	343.50	4.44359E-09
Hsd17b11	290.90	1.8856E-08	137.60	1.62911E-07
Cops7b	287.20	1.90079E-11	337.60	6.18291E-12
Dapk3	282.00	1.90079E-11	331.50	6.49299E-12
Zfp319	280.95	4.10098E-08	558.50	3.34533E-12
Atp10a	279.30	8.90659E-08	327.40	8.35991E-08
Amot	278.20	7.37541E-08	325.40	1.01172E-07
Lrrcc1	272.90	1.90079E-11	320.90	7.92423E-12
Cfap298	271.90	7.51265E-08	6.80	5.44178E-08
Cdc73	268.50	7.48258E-08	308.10	1.3055E-11
Arhgap31	268.20	1.93394E-11	315.00	1.31842E-11
Ypel2	264.50	7.98326E-08	312.60	1.08141E-07
Vti1a	261.80	8.07765E-08	306.90	8.11799E-08
Smn1	261.60	4.89905E-08	307.90	4.65058E-08
Zmiz1	259.10	1.94664E-11	2.43	1.90752E-06
Lurap1	258.20	4.31377E-08	303.00	4.86786E-08
Atp6ap2	257.80	3.73756E-08	360.70	3.90712E-08
Pcyt1a	257.30	1.08906E-07	476.90	3.18186E-08
Lrp6	254.40	5.88405E-08	298.50	4.19621E-08
Pex16	253.80	2.10765E-11	298.30	1.67794E-11
Pym1	251.10	6.68706E-08	294.20	9.20312E-08
Mon1a	249.80	5.0936E-08	293.20	3.12264E-08
Iqsec1	249.80	5.0936E-08	103.17	2.06434E-10
1810055G02Rik	249.30	2.33559E-11	293.00	1.87032E-11
Snx30	248.40	5.91467E-09	19.18	9.6349E-09
Adck2	247.70	2.07879E-10	290.90	1.06689E-10
Utrn	244.80	2.58121E-11	12.29	1.06307E-07
Veph1	238.60	2.82428E-11	489.90	2.32156E-11
Cables1	229.30	5.65575E-08	269.10	4.27357E-08

Nsmce1	227.70	9.21864E-08	331.10	7.39617E-08
Pigs	225.40	7.26122E-08	43.25	1.30136E-07
Rab3gap1	221.30	2.32223E-08	260.40	1.97551E-08
2900026A02Rik	221.30	2.32223E-08	8.33	4.54774E-11
Kif23	214.03	8.59918E-08	1435.80	2.45909E-07
Kat6b	211.51	3.90696E-10	2.20	3.3244E-05
Vps18	210.60	7.16963E-11	247.70	6.45719E-11
Entpd7	205.70	1.0329E-07	241.20	7.60598E-08
Upf3b	201.20	1.70254E-07	698.10	3.29202E-11
Isy1	192.60	3.12308E-07	227.10	9.00271E-08
Ereg	191.00	1.95599E-07	348.60	7.74275E-08
Plod1	189.80	2.00066E-07	222.40	8.77319E-08
Tmeff1	189.00	2.68638E-07	128.00	1.41145E-05
Lpp	187.2	2.0293E-07	220.4	5.76123E-08
Bmp1	187.20	2.0293E-07	2.33	2.40111E-05
Nsmf	185.40	1.4994E-08	217.70	1.78543E-09
Cecr2	185.10	3.37685E-07	166.60	4.78858E-07
Ift88	184.90	4.89702E-08	217.50	1.84049E-08
Tsc22d1	180.10	1.46876E-09	211.60	3.25044E-10
Nek4	180.00	3.78367E-07	211.00	7.62258E-08
Akap9	179.40	1.46179E-09	8.07	9.79716E-08
Fbxw7	178.56	1.39901E-10	2.11	7.3011E-05
Cit	177.60	5.02652E-07	106.00	8.1683E-05
Nrg2	177.40	6.00366E-07	24.80	2.71741E-07
Man2b2	173.80	7.01777E-07	7.71	9.267E-05
Spice1	172.20	8.82744E-07	10.12	1.79297E-05
Cwf19l2	172.10	1.58765E-06	17.98	1.51886E-11
Sel1l	166.50	6.796E-09	195.70	5.6035E-10
Gsta4	166.40	8.84352E-07	228.10	6.079E-10
Noct	161.80	4.15492E-06	18.23	3.06566E-06
Zfp462	160.20	1.94399E-06	287.50	9.88031E-08
Mrpl2	159.6	1.96099E-08	187.40	1.08935E-09
Arel1	159.50	2.83329E-06	186.80	1.1281E-07
Ube2h	153.00	6.5707E-11	652.90	2.00364E-12
Slc8b1	151.80	3.90068E-06	177.80	1.8515E-07
Fbxo10	151.10	6.30634E-06	177.00	1.78422E-07
Fam98b	150.00	6.62322E-06	176.60	1.50188E-07
Kctd17	148.45	1.14171E-07	2.02	8.91234E-06
Mpp7	148.00	5.4823E-06	173.30	2.5144E-07
Map7	145.5	7.74422E-06	10.48	5.95748E-05
Gtpbp4	139.90	2.23533E-06	164.10	8.08302E-08
Rbm19	139.70	1.1114E-05	163.50	5.29902E-07
Tradd	138.80	1.33854E-05	14.47	2.96356E-05
Trpm4	136.60	1.53215E-05	159.80	7.61186E-07

Dnajc27	135.90	1.58204E-05	403.50	8.97473E-08
Zc3h15	135.30	1.05685E-06	158.80	3.99835E-08
Dennd1a	135.30	1.05685E-06	13.58	5.79201E-05
Gss	133.50	2.04111E-05	156.00	1.08813E-06
Mboat2	131.70	2.37818E-06	154.90	1.33729E-07
Ston1	131.50	5.38262E-06	8.41	1.40273E-04
Psmc3	131.30	2.27815E-05	25.20	1.14748E-05
Riok3	130.80	3.12846E-07	153.50	3.22405E-08
Gkap1	126.20	5.26768E-07	6.96	8.94602E-05
Zfp189	125.20	4.18249E-05	146.20	2.69758E-06
Tbck	124.60	5.80077E-05	146.00	1.8245E-06
Gys1	124.10	5.26768E-07	145.80	5.77548E-08
Ncoa2	123.70	4.5799E-05	12.47	5.03349E-07
Crocc	123.00	4.73416E-05	172.50	2.48987E-07
Adk	121.7	9.10762E-07	275.70	4.01093E-11
Rtkn	121.50	4.76093E-05	142.30	3.39281E-06
Sfmbt1	118.80	3.39696E-05	139.70	1.69784E-06
Nomo1	117.90	1.59535E-06	9.11	4.69438E-05
Dazap1	113.66	2.53939E-11	788.10	2.00364E-12
Prr14	108.10	4.69126E-06	533.20	5.70051E-09
Rpa1	107.40	7.30954E-06	126.20	7.85474E-07
Atxn1	107.40	7.30954E-06	20.69	7.58814E-06
Fto	101.90	5.01585E-05	119.50	3.04076E-06
Cep89	101.20	1.46652E-05	135.40	3.8058E-06
Fbxo8	97.40	2.60836E-05	114.20	3.97608E-06
Usb1	96.70	2.60836E-05	19.89	6.24161E-05
Arl13b	96.51	1.27274E-07	5.04	3.2138E-11
Ammecr1	92.78	1.77151E-07	794.20	1.8947E-07
Add3	92.10	4.79589E-05	122.50	1.07008E-06
Armc1	88.73	2.33559E-11	653.50	5.04625E-10
Erp44	78.91	5.8984E-11	1002.30	9.7947E-12
Knstrn	74.76	1.5779E-07	1006.60	8.57347E-10
Nsun2	51.05	1.39105E-07	353.10	8.65259E-08
Sh2d4a	50.53	1.40403E-07	640.60	9.11036E-08
Tln1	45.76	4.48425E-07	1458.90	5.03506E-11
Arnt2	43.88	8.4742E-08	11.00	8.32118E-08
Helz	34.45	5.19202E-11	8.66	1.05726E-08
Trim41	34.20	3.22325E-11	11.39	5.86442E-07
Mta3	32.90	1.69846E-06	228.60	7.65689E-08
Jade1	31.55	2.15544E-07	9.29	1.27247E-06
Arhgef4	27.99	3.46652E-09	16.67	1.73137E-09
Sbf2	25.73	8.5855E-09	308.90	1.36943E-11
Trip12	25.61	4.25924E-10	2.29	2.63705E-05
Micu2	24.31	2.54829E-08	483.80	1.65712E-09

Tcof1	22.11	1.07467E-08	927.30	1.39814E-08
Rcor3	21.56	3.1929E-07	817.40	2.19732E-07
Setbp1	21.40	2.39794E-05	182.50	1.69991E-07
Tle3	21.25	2.01207E-07	19.93	3.65577E-11
Celsr2	20.83	2.43454E-11	618.20	2.00364E-12
Ttll7	20.33	2.09679E-11	140.10	4.72336E-06
Hspb8	19.78	3.08483E-07	483.2	9.67158E-08
Ahdc1	19.43	6.57686E-11	21.17	2.76932E-11
Kdm4b	18.72	1.19324E-08	571.70	1.37732E-09
Itpripl1	18.56	3.98853E-05	128.60	5.65746E-07
Fam110a	17.82	2.25862E-07	15.66	4.4513E-07
Syngap1	16.47	1.37881E-05	934.90	9.18492E-08
Ptgfrn	16.27	2.63315E-07	4.28	2.05954E-05
Atp2c1	12.76	4.02666E-11	5.27	6.29841E-06
Zfp36	11.93	3.97014E-07	1904.40	6.83858E-11
Pidd1	11.55	7.13609E-07	268.30	3.0443E-08
Dcaf5	10.88	1.97692E-07	491.80	6.60882E-08
Wfs1	10.86	7.55782E-09	408.40	2.00364E-12
Fam110c	10.28	2.52829E-08	665.30	2.15146E-09
Osbpl5	10.28	3.50346E-07	123.50	2.23424E-05
Smc3	10.13	7.59631E-08	2.42	5.3774E-07
Pdlim7	9.95	1.93394E-11	35.60	8.29449E-12
Myo1e	9.90	7.26139E-07	2.32	4.76647E-08
Prkaca	9.56	5.38885E-07	2.37	2.64189E-06
Rmdn3	9.53	1.90179E-11	6.34	1.92627E-11
Creb5	9.38	5.07522E-08	4.46	1.37016E-06
Lsm6	9.32	6.3302E-06	9.19	8.51656E-07
Naif1	9.23	2.11425E-07	417.00	4.60433E-08
Dot1l	9.08	1.03408E-07	24.43	5.03732E-08
Stk39	8.81	3.86733E-07	449.60	9.4442E-08
Fbrsl1	8.75	4.70209E-07	13.36	5.15854E-08
Tbcel	8.53	7.20325E-06	388.00	1.06307E-07
Pafah1b2	8.25	4.02918E-11	2.68	3.03388E-05
Limk2	8.10	7.83629E-08	16.86	4.30756E-07
Golga4	8.01	5.5174E-07	950.80	9.61652E-08
Peak1	7.90	3.87218E-05	183.70	1.91997E-08
Prkar2b	7.47	1.12181E-06	3.14	8.86283E-06
Sprr1a	7.46	1.68382E-07	4.18	4.11309E-07
Cep128	7.09	2.7288E-08	2.39	6.50019E-05
Emx2	6.96	1.29964E-06	3.84	1.56875E-05
Usp8	6.81	2.35784E-06	283.20	2.49122E-11
Pip5k1c	6.47	6.28496E-08	4.26	9.56883E-05
Carhsp1	6.44	3.08312E-05	4.38	3.58972E-06
Plxnb1	6.08	3.16827E-07	2.58	1.92269E-05

Col18a1	6.01	4.68022E-10	17.44	9.77827E-09
Ccdc85b	5.60	5.1652E-09	2.44	5.22692E-06
Tbl2	5.18	4.6306E-08	2.24	1.25092E-04
Plekhh2	5.11	1.16954E-07	2.15	5.49776E-05
Aptx	5.09	5.22237E-07	270.48	7.92291E-11
Cdc42bpb	4.92	5.79991E-07	2.65	4.94433E-06
Rnf6	4.72	4.27661E-07	717.90	2.00364E-12
Mapk8ip3	4.54	8.20548E-08	4.25	3.04393E-07
Sap130	4.25	3.04573E-06	258.70	9.12241E-08
Men1	4.12	2.1685E-06	18.30	4.66838E-11
Arid4b	4.08	5.15643E-05	8.10	1.26238E-11
Asph	4.04	4.55964E-06	2.56	3.12475E-05
Dab2	3.69	6.28724E-06	293.20	3.12264E-08
Trim11	3.56	2.26273E-05	13.02	4.03069E-07
Smarca2	3.41	2.04843E-06	194.90	5.32001E-10
Hint1	3.38	3.08908E-06	2.64	1.69545E-05
Mrtfb	3.24	2.34425E-06	30.78	2.00553E-07
B930094E09Rik	3.22	3.02484E-05	814.40	2.73158E-12
Rad21	3.09	3.08323E-05	1838.80	1.99982E-07
Parm1	3.08	2.60058E-06	3.11	4.10035E-06
Rpn2	3.00	4.40324E-06	4.51	1.21953E-09
Rp9	2.93	3.73096E-05	2.11	3.49217E-05
Anapc5	2.88	2.01624E-05	135.70	1.04331E-09
Bicra	2.82	1.79355E-06	3.64	2.74917E-07
Ntn1	2.75	1.38563E-05	6.30	2.75071E-05
Hk1	2.64	2.91058E-05	5638.00	7.44313E-10
Fgd1	2.54	1.75516E-07	339.90	6.79327E-11
Ube2e3	2.48	2.86088E-05	10.94	6.06418E-05
Ggnbp2	2.40	2.14252E-05	966.10	7.92423E-12
Rbmx2	2.35	8.08644E-06	1470.30	5.16482E-11
Mxi1	2.34	2.63422E-09	2.42	4.4513E-07
Csnk1e	2.32	2.63354E-05	12.01	4.30852E-07
Ubn1	2.22	4.37049E-07	618.40	2.90372E-12
Myh10	2.15	1.8232E-06	7.23	8.44932E-05
Ctdspl2	2.13	1.69551E-05	2.12	3.50562E-05
Arid1a	2.11	4.2604E-05	2.24	9.4951E-05
Lhfp	2.06	1.2252E-05	3.02	1.98097E-06

Supplementary Table 3. Primers for qRT-PCR, RIP qPCR and CHIP qPCR related to Figure 1, Figure 3, Figure 7, and STAR Methods

	Forward primer (5’-3’)	Reverse primer (5’-3’)
**Primers for qPCR**		
Nat10(mus)	AAGACCACAACGACAGCCAGAC	TCCAGGCACAGCAGGTCATTAAG
Brd4(mus)	AAGATGCCTGATGAGCCTGAAGAG	ACTGTCGCTGTCGGAAGAACTG
Pfkm(mus)	GAATGCCGCTGTTCGCTCTACC	GCCAGACCCTCAAAGCCATCATG
β-actin (mus)	GTGCTATGTTGCTCTAGACTTCG	ATGCCACAGGATTCCATACC
**Primers for RIP qPCR**		
Brd4-site 1 (mus)	GAAGTGGTAGCCATGGCTCGA	CTGCAAGCTGCTCATGCACG
Pfkm -site 1 (mus)	TCTGGTAACCAGGCTGTGCG	CTCCCAGTTGTTCATGAAGCTCC
**Primers for CHIP RT-PCR**		
Nat10-site1 (mus)	TGCCAGCACCTACACTTGTG	ACACACGTAACACACCATCT
Nat10-site2 (mus)	GCCTGTGAGTTGCATACACCC	CAAGGCTATGCAGGAAACTGCTC
Nat10-site3 (mus)	CCTAGATGGTGTGTTACGTGTGT	GCCCTACACTTTGGGTAGAAGCC
Notch1-site1(mus)	TGGTCTCGGCTATAGGCATC	GGCACTAGTCCTTGGGGAGT
